# SRY-Box transcription factor 9 triggers YAP nuclear entry via direct interaction in tumors

**DOI:** 10.1038/s41392-024-01805-4

**Published:** 2024-04-24

**Authors:** Hui Qian, Chen-Hong Ding, Fang Liu, Shi-Jie Chen, Chen-Kai Huang, Meng-Chao Xiao, Xia-Lu Hong, Ming-Chen Wang, Fang-Zhi Yan, Kai Ding, Ya-Lu Cui, Bai-Nan Zheng, Jin Ding, Cheng Luo, Xin Zhang, Wei-Fen Xie

**Affiliations:** 1 Department of Gastroenterology, Changzheng Hospital, Naval Medical University, Shanghai, China; 2 Department of Gastroenterology, Shanghai East Hospital, School of Medicine, Tongji University, Shanghai, China; 3 Drug Discovery and Design Center, CAS Key Laboratory of Receptor Research, State Key Laboratory of Drug Research, Shanghai Institute of Materia Medica, Chinese Academy of Sciences, Shanghai, China; 4 Department of Gastroenterology, First Affiliated Hospital of Nanchang University, Nanchang, China; 5 Clinical Cancer Institute, Center for Translational Medicine, Naval Medical University, Shanghai, China

**Keywords:** Drug development, Oncogenes

## Abstract

The translocation of YAP from the cytoplasm to the nucleus is critical for its activation and plays a key role in tumor progression. However, the precise molecular mechanisms governing the nuclear import of YAP are not fully understood. In this study, we have uncovered a crucial role of SOX9 in the activation of YAP. SOX9 promotes the nuclear translocation of YAP by direct interaction. Importantly, we have identified that the binding between Asp-125 of SOX9 and Arg-124 of YAP is essential for SOX9-YAP interaction and subsequent nuclear entry of YAP. Additionally, we have discovered a novel asymmetrical dimethylation of YAP at Arg-124 (YAP-R124me2a) catalyzed by PRMT1. YAP-R124me2a enhances the interaction between YAP and SOX9 and is associated with poor prognosis in multiple cancers. Furthermore, we disrupted the interaction between SOX9 and YAP using a competitive peptide, S-A1, which mimics an α-helix of SOX9 containing Asp-125. S-A1 significantly inhibits YAP nuclear translocation and effectively suppresses tumor growth. This study provides the first evidence of SOX9 as a pivotal regulator driving YAP nuclear translocation and presents a potential therapeutic strategy for YAP-driven human cancers by targeting SOX9-YAP interaction.

## Introduction

The translocation of Yes-associated protein (YAP) from the cytoplasm to the nucleus is a central cellular event necessary for its function of promoting aberrant cell growth and tumorigenesis.^1^ Extensive research has been conducted to elucidate the mechanisms regulating the cytoplasmic sequestration and nuclear retention of YAP. In the cytoplasm, YAP interacts with a group of proteins, including 14-3-3, AMOT, PTPN14, and α-catenin, which are responsible for its cytoplasmic sequestration.^2–5^ Conversely, the direct binding of YAP with its nuclear partners, including TEADs and MAML1/2, promotes the nuclear retention of YAP, thereby stimulating cell proliferation, survival, and migration, and driving drug resistance.^6,7^ Maintaining the delicate balance between cytoplasmic sequestration and nuclear retention is crucial for YAP function. In addition, YAP contains a conventional nuclear export signal, which facilitates its nuclear export.^8^ However, unlike its *Drosophila* ortholog *Yorkie*, no classical nuclear localization signal (NLS) has been identified in mammalian YAP proteins.^9^ The absence of a canonical NLS poses an intriguing question about the mechanisms governing the nuclear import of YAP in mammalian cells. Although the force applied to the nucleus increases YAP nuclear import by reducing the mechanical restriction of nuclear pores,^10^ further molecular efforts are still warranted to unravel the mechanisms of YAP nuclear import and its potential implications in disease pathogenesis.

SRY-related high-mobility group box 9 (SOX9) acts as a transcription factor that plays a pivotal role in various biological processes, including stemness, sex determination, and progenitor development.^11^ SOX9 has been widely characterized as a candidate cancer stem cell marker. The involvement of SOX9 in oncogenesis, chemoresistance, and stem cell maintenance in cancers highlights its critical regulatory functions in tumor biology.^12^ YAP and SOX9 are aberrantly upregulated in many tumors, where they serve as critical regulators driving tumor progress.^13,14^ YAP transcriptionally promotes SOX9 expression in the development of several tumors,^15,16^ whereas the role and mechanism of SOX9 in the regulation of YAP activity remains controversial. It has been demonstrated that SOX9 represses YAP expression by increasing the level of YAP-targeting microRNAs in esophageal squamous cell carcinoma.^16^ Recent studies indicated that SOX9 may disrupt the concerted interactions between YAP/TEAD and alter the response elements of genes targeted by YAP in hepatocytes.^17^ In the contrary, SOX9 has been found to increase YAP activity and promote the epithelial-mesenchymal transition (EMT) in gastric carcinoma.^18^ This finding suggests that SOX9 may act as a positive regulator of YAP activity, depending on the cellular microenvironment and the tumor heterogeneity. In addition, while the dynamic activity of YAP is dependent on its relative level of nuclear import,^19^ whether SOX9 affects the nuclear translocation of YAP remains unexplored.

Posttranslational modification (PTM) is an essential regulatory mechanism engaged in the nucleoplasm distribution of YAP.^20^ The phosphorylation of Ser-127 is mediated by Hippo pathway, which is a crucial factor in determining the subcellular localization of YAP. This process serves as a binding site for 14-3-3 proteins, leading to the sequestration of YAP in the cytoplasm and inhibition of its target gene activity.^21^ Another PTM that impacts the subcellular localization of YAP is monomethylation at lysine 342 (K342) mediated by SET1A. This modification of YAP blocks CRM1-mediated nuclear export of YAP, leading to its accumulation in the nucleus of multiple tumor cells.^8^ PRMT1 is the major member of the type I protein arginine methyltransferases (PRMTs) family. PRMT1 selectively catalyzes the asymmetric dimethylarginine (Rme2a, also known as ADMA) modification at arginine residue of substrate proteins.^22^ Previous studies have revealed that PRMT1 exhibits abnormal expression in tumor cells and methylates diverse substrate proteins involved in the aggressiveness, drug sensitivity, and prognostic judgment of cancers.^23,24^ Recently, it has been verified that arginine methylation by PRMT1 affects the subcellular localization and turnover of p14 (ARF).^25^ Of note, it has been reported that there is a positive association between elevated PRMT1 level and YAP nuclear accumulation in chondrosarcoma specimens.^26^ Nevertheless, whether specific arginine methylation of YAP is responsible for its subcellular localization has not been reported.

In the current study, we observed a significant reduction in YAP’s transcriptional activity and nuclear fraction in cancer cells where SOX9 was either knocked out or knocked down. We confirmed a direct interaction between SOX9 and YAP both in vitro and in vivo. Furthermore, our research elucidates the mechanism by which exogenous SOX9 facilitates the translocation of YAP from the cytoplasm into the nucleus, relying on the specific interaction between Asp-125 in SOX9 and Arg-124 in YAP. We also found that PRMT1-mediated asymmetrical dimethylation of YAP at R124 enhances the binding between YAP and SOX9, leading to the accumulation of YAP in the nucleus. Moreover, we discovered that high levels of YAP-R124me2a, but not YAP alone, were predictive of poor prognosis in multiple cancers. Based on these findings, we developed a selective peptide that mimics the fragment of SOX9 containing D125 (111-126a.a.), disrupting the interaction between YAP and SOX9 and specifically inhibiting YAP-driven HCC growth. Our study sheds light on the role of SOX9 in the translocation of YAP into the nucleus and provides insights into the underlying mechanism.

## Results

### SOX9 is pivotal for YAP activity in HCC cells

**Fig. 1:**
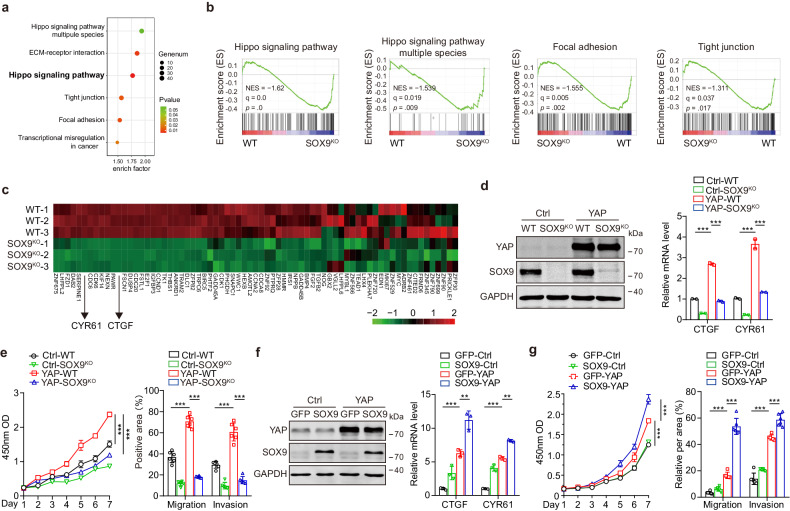
SOX9 is pivotal for YAP activity in HCC cells. **a** Analysis of the KEGG pathway associated with differentially expressed genes between Huh-7 and SOX9^KO^ Huh-7 cells. The criteria for differentially expression were a significant q value (q < 0.05) and fold-change thresholds, with a 2-fold increase indicating upregulation and a 0.5-fold decrease indicating downregulation. **b** GSEA of the Hippo pathway, the Hippo pathway-multiple species, focal adhesion pathway, and tight junction pathway using RNA-seq data obtained from Huh-7 and SOX9^KO^ Huh-7 cells. **c** Heatmap of YAP-target gene expression profiles based on RNA-seq data. **d** Huh-7 and SOX9^KO^ Huh-7 cells were infected with lenti-YAP or control virus (Ctrl), and the YAP and SOX9 expression levels were detected by immunoblotting (left); the *CTGF* and *CYR61* mRNA levels were examined by qPCR (right), data are represented as mean ± SD, ***P < 0.001. **e** The proliferation capacity (left), and migration and invasion capacity of the cells described in d were measured (right), data are represented as mean ± SD, ***P < 0.001. **f** YAP and SOX9 were upregulated in Huh-7 cells by lentivirus and adenovirus, and the YAP and SOX9 expression levels were detected by immunoblotting (left); qPCR analysis of *CTGF* and *CYR61* in Huh-7 cells (right), data are represented as mean ± SD, **P < 0.01. ***P < 0.001. **g** The proliferation capacity (left), and migration and invasion capacity of the cells described in f were measured (right), data are represented as mean ± SD, ***P < 0.001

To assess whether SOX9 affects YAP activation, SOX9 knockout (SOX9^KO^) Huh-7 cells were established using the CRISPR-Cas9 system. RNA sequencing (RNA-seq) data revealed global suppression of known YAP-related pathways and YAP-targeted genes in SOX9^KO^ cells (Fig. 1a–c). Real-time PCR analysis verified that depletion of SOX9 in Huh-7 cells not only inhibited the expression of the SOX9-target genes *OPN* and *COL2A1* but also decreased the mRNA levels of the YAP-target genes *CYR61* and *CTGF* (supplementary Fig. 1a). To determine the effect of SOX9 on YAP-driven malignancy, we infected hepatocellular carcinoma (HCC) cells with lenti-YAP. The mRNA levels of *CYR61* and *CTGF* induced by YAP were also downregulated in SOX9^KO^ Huh-7 cells compared with Huh-7 cells (Fig. 1d). Consistent with these observations, the depletion of SOX9 obviously inhibited the proliferation, migration, and invasion of Huh-7 cells expressing YAP (Fig. 1e; supplementary Fig. 1b). In addition, the knockdown of SOX9 by siRNA against SOX9 also notably attenuate the YAP-induced gene expression and malignant features of PLC cells (supplementary Fig. 1c, d).

To evaluate the potential synergistic effect of YAP and SOX9 on HCC malignancy, the expression of YAP and SOX9 in HCC cells were upregulated by lentivirus and adenovirus, respectively. Elevated expression of SOX9 significantly enhanced the transcription of YAP-targeted genes in HCC cells (Fig. 1f; supplementary Fig. 1e). Moreover, the combined administration of lenti-YAP and Ad-SOX9 significantly increased the oncogenic properties of HCC cells comparing to the treatment of either lenti-YAP or Ad-SOX9 (Fig. 1g; supplementary Fig. 1f, g). Taken together, these results identified SOX9 as a novel and potent modulator of YAP function during HCC progression.

### SOX9 triggers the nuclear translocation of YAP via direct interaction

**Fig. 2:**
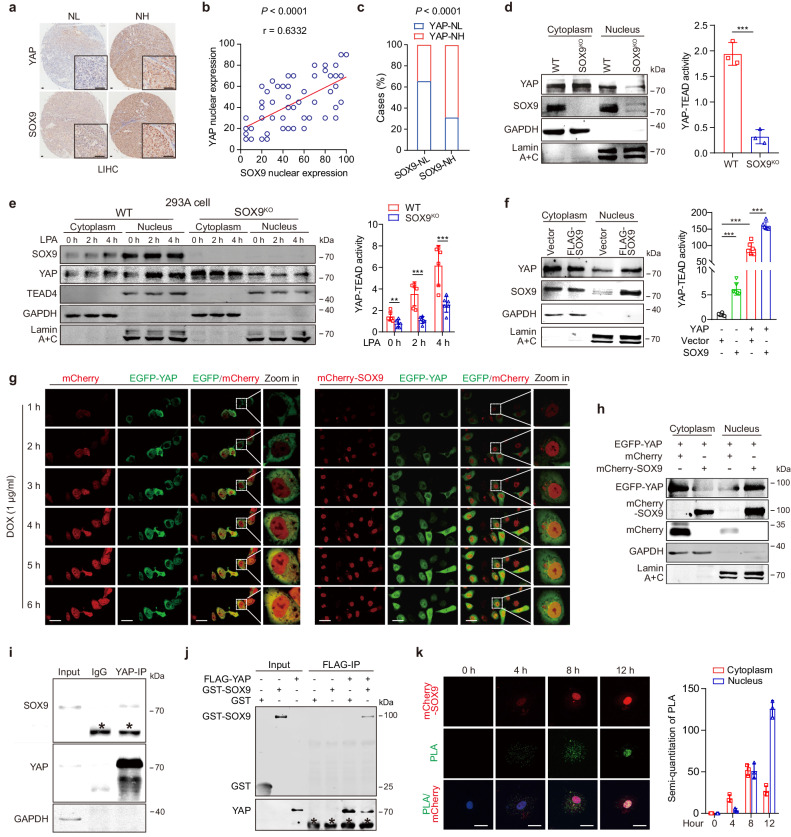
SOX9 triggers the nuclear translocation of YAP via direct interaction. **a** Representative image of YAP and SOX9 immunohistochemistry (IHC) staining of human hepatocellular carcinoma (LIHC) tumors. NL (nuclear-low) and NH (nuclear-high) indicate the nuclear levels of SOX9 and YAP. The cut-off value for the levels of YAP or SOX9 in the nucleus is the median. Scale bar = 100 μm. **b** Correlation of the nuclear levels of SOX9 and YAP in tissue microarray (TMA) of human hepatocellular carcinoma (LIHC) (*n* = 64). The statistical method for analyzing the nuclear level of proteins is described in Methods. Data are represented as mean ± SD. **c** Tissues of the patients in TMA were divided into two groups according to nuclear levels of SOX9, then further grouped into YAP nuclear-high (NH) expression and nuclear-low (NL) expression according to nuclear levels of YAP. The percentage of patients in different groups is shown. The median value of YAP or SOX9 in the nucleus was utilized as the cutoff point to categorize patients. **d** Nuclear and cytoplasmic fractions of Huh-7 cells and SOX9^KO^ Huh-7 cells were separated and followed by immunoblot assay to evaluate the YAP and SOX9 expression level (left); The transcriptional activity of YAP-TEAD was examined using the 8×GTIIC reporter in Huh-7 cells and SOX9^KO^ Huh-7 cells (right). Data are represented as mean ± SD, ***P < 0.001. **e** The protein levels of SOX9, YAP, and TEAD4 in the cytoplasmic and nuclear fractions of 293 A cells and SOX9^KO^ 293 A cells after LPA treatment for the indicated times (left); The 8×GTIIC reporter activity in 293 A cells and SOX9^KO^ 293 A cells treated with LPA for the indicated times (right). Data are represented as mean ± SD, **P < 0.01, ***P < 0.001. **f** Nuclear and cytoplasmic fractions were separated and followed by immunoblot assay to access the YAP expression level in Huh-7 cells co-transfected with YAP plasmid and control (Vector) or SOX9 plasmid (left). The 8×GTIIC reporter activity in Huh-7 cells co-transfected with YAP plasmid and control (Vector) or SOX9 plasmid (right). Data are represented as mean ± SD, ***P < 0.001. **g** Live cell-imaging showing the nuclear influx of EGFP-YAP in Huh-7 cells infected with lentiviruses expressing DOX-inducible mCherry-SOX9 and EGFP-YAP reporter fusion proteins. The expression of mCherry-SOX9 and EGFP-YAP was induced by 1 μg/ml DOX. Scale bar = 20 μm. **h** Nuclear and cytoplasmic fractions were separated and followed by immunoblot assay to access the expression of YAP in Huh-7 cells with DOX-inducible expression of EGFP-YAP and mCherry-SOX9 after treatment with DOX for 24 h. **i** Co-immunoprecipitation was performed with extracts from Huh-7 cells. * indicates heavy chain. **j** In vitro protein pull-down assay performed with recombinant GST-SOX9 and FLAG-YAP using anti-FLAG beads. * indicates heavy chain. **k** Representative images of PLA using antibodies against SOX9 and YAP in different cells (left). Green spots indicate a YAP/SOX9 protein interaction, and DAPI-stained nuclei are blue. The quantification of green spots in the cytoplasm and nucleus was performed using ImageJ software (right). Scale bar = 20 μm

It is known that the Hippo pathway inhibits YAP activation via LATS-mediated phosphorylation of YAP.^27^ Here, we found that knockout of SOX9 slightly reduced the level of LATS1 and barely altered the expression and the phosphorylation of YAP in Huh-7 cells (supplementary Fig. 2a), suggesting that SOX9 may regulate YAP activity in a Hippo pathway-independent manner. To investigate the underlying mechanism by which SOX9 regulates YAP function, we first detected the expression of SOX9 and YAP in HCC tissues (Fig. 2a). The nuclear level of YAP was positively correlated with that of SOX9 in HCC samples (*P* < 0.0001, *r* = 0.6332, *n* = 64, Fig. 2b). Obvious YAP nuclear localization was observed in 69% (22/32) of cases with higher levels of SOX9 but in only 34% (11/32) of cases with low levels of SOX9 (*P* < 0.0001, Fig. 2c). This finding prompted us to investigate the effect of SOX9 on YAP subcellular localization. Depletion of SOX9 reduced the nuclear levels of YAP and YAP-TEAD transcriptional activity in Huh-7 cells (Fig. 2d, supplementary Fig. 2b–d). Moreover, knockout of SOX9 also dramatically inhibited lysophosphatidic acid (LPA)-induced YAP nuclear translocation and YAP-TEAD transcriptional activity in HEK-293A cells (Fig. 2e). On the contrary, the overexpression of SOX9 significantly enhanced YAP activity and increased the nuclear levels of YAP in HCC cells and HEK-293A cells. (Fig. 2f; supplementary Fig. 2e–g).

To further evaluate SOX9’s function in promoting YAP nuclear translocation, Huh-7 cells were infected with lentivirus expressing doxycycline (DOX)-inducible mCherry-SOX9 and EGFP-YAP. Both EGFP-YAP and mCherry-SOX9 were simultaneously expressed upon the addition of DOX. Nuclear translocation of EGFP-YAP was detected in the cells co-expressing mCherry-SOX9, whereas little nuclear EGFP-YAP was observed in the cells with control mCherry expression at 6 h after DOX induction (Fig. 2g). Furthermore, mCherry-SOX9 promoted the translocation of EGFP-YAP into nuclei in Huh-7 cells at 24 h after DOX induction (Fig. 2h). Together, these data indicate that SOX9 is required for the nuclear translocation of YAP.

Co-immunoprecipitation (Co-IP) assay showed that SOX9 interacted with YAP but did not obviously bind TEAD4 (Fig. 2i; supplementary Fig. 3a). In vitro pull-down assay demonstrated the direct interaction between SOX9 and YAP (Fig. 2j). In situ proximity ligation assays (PLAs) conducted in HEK-293A cells and various tumor cells confirmed YAP-SOX9 interaction in both the cytoplasm and the nucleus (supplementary Fig. 3b, c), whereas the depletion of SOX9 in Huh-7 cells completely diminished the fluorescence signal (supplementary Fig. 3d). These data prompted us to speculate whether SOX9 promotes the nuclear translocation of YAP. To verify this, we conducted Co-IP with the cytoplasmic and nuclear protein fractions extracted from Huh-7 cells that were transiently transfected with SOX9. The interaction of exogenous SOX9 and YAP was first observed in cytoplasm and then gradually detected in the nuclei along with the nuclear import of exogenous SOX9 (supplementary Fig. 3e). PLAs also confirmed that the aggregation of endogenous YAP and exogenous SOX9 gradually shifted from the cytoplasm to the nucleus in cells infected with lentivirus expressing DOX-inducible mCherry-SOX9 (Fig. 2k; supplementary Fig. 3f). Collectively, these results support the hypothesis that the direct interaction between SOX9 and YAP promotes the nuclear transport of YAP.

### The Asp-125 residue of SOX9 is required for SOX9-induced YAP nuclear translocation

**Fig. 3:**
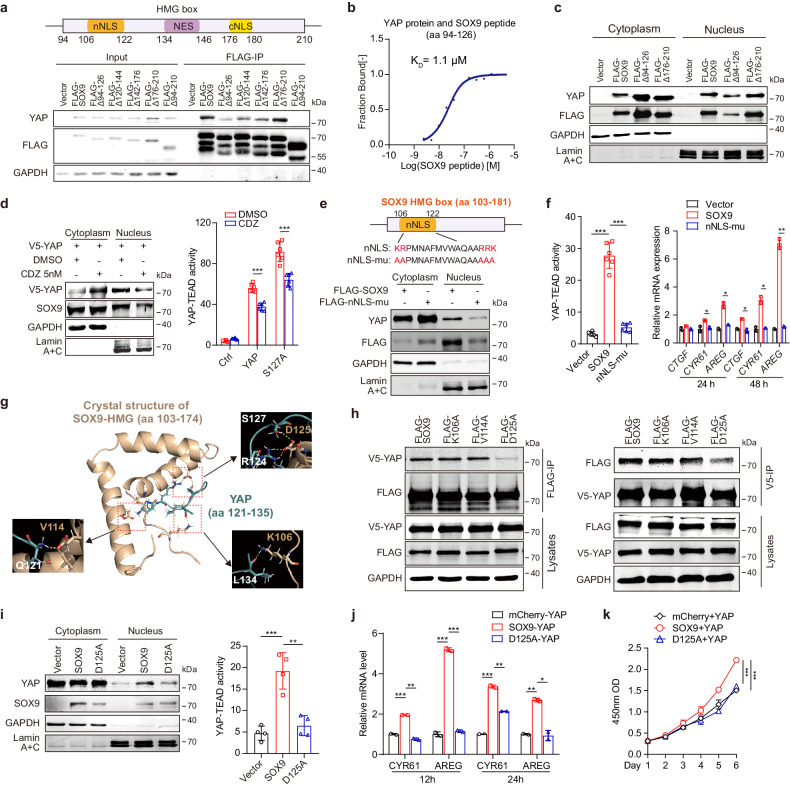
The Asp-125 residue of SOX9 is required for SOX9-induced YAP nuclear translocation. **a** Map of the HMG domain of the SOX9 protein (upper). Huh-7 cells were transfected with different truncations of the SOX9-HMG domain (Δ94–126, Δ120–144, Δ142–176, Δ176–210, Δ94–210). FLAG-IP was performed with the cell extracts, followed by immunoblotting analysis. **b** MST assay to determine the binding affinity between the YAP protein and nNLS-containing fragment of SOX9 (aa 94–126). **c** Nuclear and cytoplasmic fractions were separated and followed by immunoblot assay to access the expression level of YAP in Huh-7 cells transfected with FLAG-SOX9 or its truncations (Δ94–126, Δ176–210). **d** Nuclear and cytoplasmic fractions were separated and followed by immunoblot assay to access the expression level of YAP in Huh-7 cells transfected with V5-YAP upon CaM inhibitor CDZ treatment for 24 h (right); The 8×GTIIC reporter activity in Huh-7 cells infected with lenti-YAP, lenti-S127A, or control lentivirus, followed by treatment with CDZ or DMSO for 24 h (right). Data are represented as mean ± SD, ***P < 0.001. **e** Schematic showing the nNLS mutant sequence (nNLS-mu, upper); Immunoblot assay was conducted to evaluate the expression level of YAP in the cytoplasmic and nuclear fractions of Huh-7 cells transfected with FLAG-SOX9 or FLAG-nNLS-mu (lower). **f** The 8×GTIIC reporter activity in Huh-7 cells transfected with SOX9, nNLS-mu, or control plasmid for 24 h (left). Data are represented as mean ± SD, ***P < 0.001; q*P*CR analysis of *CTGF*, *CYR61,* and *AREG* expression levels in Huh-7 cells transfected with SOX9, nNLS-mu or control plasmid for 24 h or 48 h (right). Data are represented as mean ± SD, *P < 0.05, **P < 0.01. **g** Putative binding model of residues 121–135 of YAP (colored in teal) and the SOX9-HMG domain (PDB ID 4EUW, colored in wheat). The key residues forming hydrogen bonds are marked and represented as sticks. Hydrogen bonds are depicted as yellow dashed lines. The predicted structure of YAP (121–135) was downloaded from the AlphaFold Protein Structure Database (AF-P46937-F1, https://alphafold.ebi.ac.uk/entry/P46937). More details of generating the docking model figure are described in Methods section. **h** Huh-7 cells were co-transfected with the V5-YAP plasmid and FLAG-tagged SOX9 or SOX9 with mutations (K106A, V114A, or D125A). Immunoprecipitation was performed on cell extracts using anti-FLAG beads (left) or anti-V5 beads (right), followed by immunoblot analysis. **i** Immunoblot analysis of the cytoplasmic and nuclear fractions of YAP in SOX9^KO^ cells with the overexpression of SOX9 and SOX9-D125A for 24 h (left); The 8×GTIIC reporter activity of SOX9^KO^ cells treated as described (right). Data are represented as mean ± SD, **P < 0.01, ***P < 0.001. **j** qPCR analysis of *CYR61* and *AREG* expression in Huh-7 cells with DOX-inducible EGFP-YAP and mCherry fusion proteins Data are represented as mean ± SD, *P < 0.05, **P < 0.01, ***P < 0.001. **k** The proliferation of Huh-7 cells with DOX-inducible co-expression of EGFP-YA*P* and mCherry fusion proteins. Data are represented as mean ± SD, ***P < 0.001

To clarify the molecular basis of the interaction between SOX9 and YAP, deletion mutants of SOX9 were established and Co-IP assay revealed that the fragment of SOX9 containing an HMG domain (amino acids [aa] 94–210) was involved in the interaction of YAP and SOX9 (supplementary Fig. 4a). The direct binding of SOX9-HMG (aa 98–181) with YAP was validated by microscale thermophoresis (MST) assay (*K*_D_ = 0.54 μM, supplementary Fig. 4b). The HMG domain of SOX9 contains two NLSs, namely the N-terminal NLS (nNLS, aa 106–122) and C-terminal NLS (cNLS, aa 175–180), which ensure the nuclear translocation of SOX9.^28^ Co-IP assay showed that deletion of the nNLS-containing fragment (Δ94–126) significantly impaired the binding of SOX9 to YAP, while deletion of the cNLS-containing fragment (Δ176–210) only slightly affected their interaction (Fig. 3a). MST assay further confirmed the high-affinity interaction of aa 94–126 of SOX9 with YAP (*K*_D_ = 1.1 μM, Fig. 3b). PLA also showed that deletion of this region (Δ94–126) abolished the aggregation of exogenous SOX9 with YAP (supplementary Fig. 4c). Deletion of SOX9-HMG or aa 94–126 significantly reduced the nuclear levels of both SOX9 and YAP (Fig. 3c; supplementary Fig. 4d), whereas deletion of aa 176–210 had no such effect (Fig. 3c).

We then evaluated the role of SOX9-nNLS in the nuclear transport of YAP. It is known that the SOX9-nNLS mediates the nuclear localization of SOX9 through the calcium-binding protein calmodulin (CaM).^29^ We found that calmidazolium chloride (CDZ, 5 nM), a CaM antagonist, not only decreased the nuclear level of SOX9, but also significantly inhibited nuclear translocation of YAP and the transactivation of the YAP-TEAD reporter upon overexpression of either YAP or YAP^S127A^ (Fig. 3d). As SOX9-nNLS is a typical bipartite NLS,^30^ we generated a mutant of SOX9 (nNLS-mu) by replacing the key residues of SOX9-nNLS (K106, R107, R120, R121, and K122) with Ala (Fig. 3e). nNLS-mu notably impeded the cytoplasm-to-nucleus translocation of both SOX9 and YAP (Fig. 3e), and abolished the promoting effect of SOX9 on the transactivation of the YAP-TEAD reporter and YAP-target genes (Fig. 3f), suggesting that nNLS of SOX9 is required for SOX9-driven YAP nuclear entry.

We next analyzed the YAP motif responsible for its binding with SOX9. Co-IP experiments showed that aa 115–135 of YAP were indispensable for the interaction of YAP and SOX9 (supplementary Fig. 4e). We then predicted the structure of the YAP (aa 121–135)-SOX9 complex interface according to the crystal structure of the SOX9-HMG fragment (aa 103–174) using the Glide program in Schrödinger.^31^ The predicted binding mode showed that the hydrogen bonding interactions between the YAP peptide (aa 121–135) and SOX9-HMG: the hydrogen bonds may be formed between K106 of SOX9 and L134 of YAP, V114 of SOX9 and Q121 of YAP, and D125 of SOX9 and R124, S127 of YAP, respectively (Fig. 3g). Reciprocal co-IP experiments showed that the D125A mutation almost completely abolished the interaction of SOX9 and YAP, while the K106A/V114A mutations had no such impact (Fig. 3h). The key role of the D125 residue of SOX9 in SOX9-YAP binding was further confirmed by MST in vitro (supplementary Fig. 4f). We next questioned whether D125 of SOX9 affects the nucleocytoplasmic localization of either SOX9 or YAP. The D125A mutation barely altered the nuclear localization of SOX9, but it decreased the SOX9-induced nuclear translocation and activity of YAP in Huh-7 cells (Fig. 3i). Moreover, live cell-imaging analysis showed that the D125A mutation only blocked the nuclear entry of EGFP-YAP without affecting the subcellular localization of mCherry-SOX9 (supplementary Fig. 4g). Furthermore, the effects of SOX9 on YAP-induced transactivation and the proliferation of HCC cells were also notably decreased by D125A mutation (Fig. 3j, k, supplementary Fig. 4h-j). Taken together, these results indicated that the D125 residue is required for the SOX9-YAP interaction and further confirmed that SOX9 is a nuclear shuttle of YAP.

### The Arg-124 residue of YAP participates in SOX9-triggered YAP nuclear translocation

**Fig. 4:**
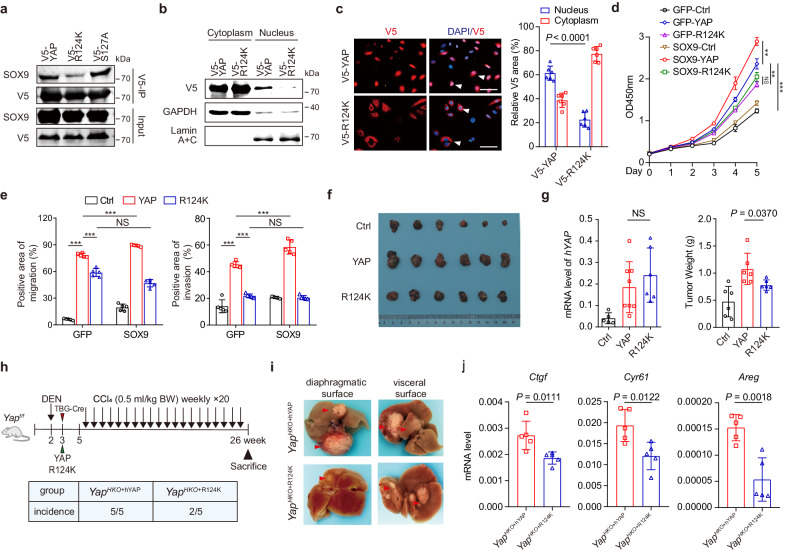
The Arg-124 residue of YAP mediates SOX9-dependent YAP nuclear localization. **a** Immunoprecipitation was performed with extracts from Huh-7 cells transfected with V5-tagged YAP or YAP with mutations (R124K, S127A) using anti-V5 beads, followed by immunoblot analysis. **b** The cytoplasmic and nuclear fractions were separated. And subsequent immunoblot analysis with V5 antibody showed the change of YAP protein in subcellular location of Huh-7 cells infected with lenti-YAP or lenti-R124K. **c** Immunofluorescence staining of V5-YAP in Huh-7 cells described in b (left). The quantification of red staining in the cytoplasm and nucleus was performed using ImageJ software (right). Scale bar = 50 μm. **d** The proliferation of Huh-7 cells infected with lenti-Ctrl, lenti-YAP, or lenti-R124K and co-infected with Ad-SOX9 or Ad-GFP control virus. Data are represented as mean ± SD, NS indicates not significant, **P < 0.01, ***P < 0.001. **e** The migration and invasion of Huh-7 cells described in d. Data are represented as mean ± SD, NS indicates not significant, **P < 0.01, ***P < 0.001. **f** Nude mice were inoculated in the right flank with Huh-7 cells (1×10^6^) infected with lenti-Ctrl, lenti-YAP, or lenti-R124K to induce HCC xenograft (n = 6 mice per group). Images of Huh-7 xenografts. **g** qPCRs were performed to assess the expression levels of human *YAP* (*hYAP*) in tumors originating from Huh-7 xenografts. Data are represented as mean ± SD, NS indicates not significant (left). Tumor weight of the Huh-7 xenografts. Horizontal line indicates the median value (right). Data are represented as mean ± SD. **h** Schematic outlines of the experiment design(upper). *YAP*^*f/f*^ mice were injected with DEN at week 2, followed by intravenous co-injection of AAV-TBG-Cre and AAV-TBG-YAP or AAV-TBG-YAP^R124K^ at week 3. The *YAP*^*H-KO*^ mice were then intraperitoneally injected with CCl_4_ weekly at week 5. The incidence of liver tumor formation was analyzed at week 26 (n = 5 in each group, lower). **i** Representative images of livers with multifocal tumors from mice described in h. **j** qPCR analysis of *Ctgf, Cyr61*, and *Areg* expression level in liver tissue isolated from mice described in h, data are represented as mean ± SD

Docking analysis revealed that the D125 residue of SOX9 interacts simultaneously with R124 and S127 of YAP (Fig. 3g). Of note, both residues are located in a 14-3-3 binding motif (RX1-2SX2-3S, supplementary Fig. 5a), which is well known to sequester YAP in the cytoplasm upon phosphorylation of Ser-127.^32^ This prompted us to evaluate the potential role of both residues (R124 and S127) in the interaction of YAP and SOX9. Co-IP experiments showed that the R124K mutation but not S127A notably abrogated the interaction of YAP and SOX9 (Fig. 4a). Moreover, the R124K mutation also dramatically blocked YAP nuclear entry and reduced the transactivation of the YAP-TEAD reporter despite the reduction of S127 phosphorylation of YAP and its binding with 14‐3‐3 (Fig. 4b, c; supplementary Fig. 5b–d). Consistent with these observations, mutation of R124K reduced YAP-induced malignancy as well as the oncogenic effect of YAP in Huh-7 cells (Fig. 4d, e; supplementary Fig. 5e). Furthermore, the enhancement of YAP-induced HCC malignancy was completely abolished by the R124K mutation (Fig. 4f, g; supplementary Fig. 5f).

Given that YAP^S127A^ is a constitutively activated form of YAP,^21^ we injected AAV8-TBG-YAP^S127A^ or AAV8-TBG-YAP^S127A-R124K^ into the wildtype mice to assess the role of R124K in the oncogenic effect of YAP activation in vivo. As expected, there was significantly less tumor formation and hepatomegaly in YAP^S127A-R124K^-overexpressing mice compared with those overexpressing YAP^S127A^ (supplementary Fig. 5g, h). We next used N-nitrosodiethylamine (DEN) plus carbon tetrachloride (CCl_4_) injection to induce HCC in hepatocyte-specific YAP knockout (*Yap*^*HKO*^) mice overexpressing human YAP or YAP^R124K^ (Fig. 4h). All *Yap*^*HKO+hYAP*^ mice developed visible tumors, whereas only two of five *Yap*^*HKO+R124K*^ mice developed small tumor nodules (Fig. 4h, i). Real-time PCR confirmed the similar expression levels of human *YAP* in the livers of AAV-injected mice (supplementary Fig. 5i). The R124K mutation also significantly decreased the expression of YAP downstream target genes and reduced the proliferation of tumor cells in the livers with DEN-CCl_4_ treatment (Fig. 4j; supplementary Fig. 5j). Immunohistochemistry staining showed the tumor tissues displayed typical HCC features, including the expression of GPC3 and AFP (supplementary Fig. 5j, k). Taken together, our data suggest that YAP-R124 is the key amino acid for the interaction of YAP with SOX9 and thus affects YAP oncogenic activity.

### PRMT1-mediated R124me2a modification of YAP promotes YAP nuclear entry and is associated with poor patient prognosis for multiple adenocarcinomas

**Fig. 5:**
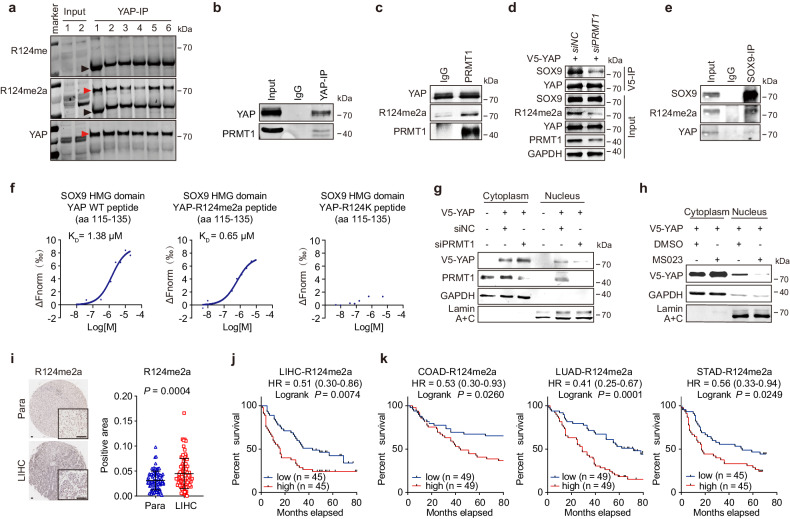
The asymmetrical dimethylation of YAP at R124 mediated by PRMT1 is associated with poor patient prognosis for multiple adenocarcinomas. **a** The levels of monomethylarginine (MMA) and asymmetrical dimethylarginine (ADMA) methylation of the YAP protein in six human HCC tissues. **b** Immunoprecipitation was performed with YAP antibody to detect the interaction between PRMT1 and YAP. **c** An in vitro methylation assay was conducted with recombinant YAP purified from SF9 cells and PRMT1 precipitated from Huh-7 cells. **d** V5-IP was performed with the extracts of Huh-7 cells co-transfected with V5-YAP and *siPRMT1*, followed by immunoblotting analysis of the interaction of SOX9 and YAP. **e** YAP-R124me2a was co-immunoprecipitated with SOX9 in Huh-7 cells. **f** The binding affinity between SOX9-HMG and unmodified YAP peptide (WT, left), methylated YAP peptide (R124me2a, middle), or mutation YAP peptide (R124k, right) was tested by MST assay. **g** Nuclear and cytoplasmic fractions of Huh-7 cells co-transfected with YAP and siNC or siPRMT1 were separated and followed by immunoblot assay to access the expression level of YAP. **h** Nuclear and cytoplasmic fractions of Huh-7 cells treated with the PRMT1 inhibitor MS023 (200 nM) or DMSO were separated and followed by immunoblot assay to access the expression level of YAP. **i** Representative images of immunohistochemical staining of R124me2a in TMA of LIHC (Scale bar = 100 μm, *n* = 90, left). Quantification of the R124me2a-positive area in cancer and para noncancerous (Para) tissue (n = 90, right). **j** Kaplan-Meier analysis was conducted to assess the overall survival of 90 patients with HCC. The median value of R124me2a expression was utilized as the cut-off point to separate patients. **k** The overall survival of patients with colorectal adenocarcinoma (COAD, left), lung adenocarcinoma (LUAD, middle), or gastric adenocarcinoma (STAD, right) according to the expression level of R124me2a

Using mass spectrometry analysis, we detected putative methylation of YAP at R124 (supplementary Fig. 6a). We then developed rabbit polyclonal antibodies that specifically recognize the three types of methylation on YAP at the R124 site. These antibodies were designed to target monomethylation (R124me1), symmetric dimethylation (R124me2s), and asymmetric dimethylation (R124me2a) (supplementary Fig. 6b). Only asymmetric dimethylation of YAP-R124 (YAP-R124me2a) was detected in HCC cells and HCC specimens (Fig. 5a; supplementary Fig. 6c, d). PRMT1 is the major member of the type I PRMT that generates asymmetric dimethylarginine. It has been demonstrated that PRMT1 expression is increased in HCC tissues, promoting the growth of HCC cells.^33^ We also found PRMT1 enhanced the malignancy of Huh-7 cells (supplementary Fig. 6e–h). Of note, Co-IP assay revealed the direct binding of PRMT1 with YAP (Fig. 5b; supplementary Fig. 6i). An in vitro methylation assay confirmed that asymmetric dimethylation of YAP-R124 was directly catalyzed by PRMT1 (Fig. 5c). Knockdown of PRMT1 decreased the YAP-R124me2a level and reduced YAP-SOX9 interaction (Fig. 5d). The interaction of YAP-R124me2a with SOX9 was also confirmed by Co-IP experiment (Fig. 5e). MST assay showed that the binding affinity of YAP peptide (aa 115–135) with SOX9-HMG domain was increased with R124me2a modification (*K*_D_ = 0.65 μM of R124me2a vs *K*_D_ = 1.38 μM of unmodified peptide) but abolished with R124K mutation (Fig. 5f), suggesting that the asymmetric dimethylation of YAP-R124 enhanced the interaction of YAP and SOX9. Consistent with this, PRMT1 overexpression increased, whereas siPRMT1 decreased YAP activity (supplementary Fig. 6j). Moreover, treatment with siPRMT1 or a PRMT1 inhibitor remarkedly blocked the nuclear transport of YAP in HCC cells (Fig. 5g, h). Furthermore, the knockdown of PRMT1 significantly reversed the elevated TEAD activity and oncogenic properties of Huh-7 cells triggered by YAP (supplementary Fig. 6k–m). Collectively, these data demonstrate that PRMT1-mediated asymmetric dimethylation of YAP-R124 increases YAP activity by promoting its nuclear translocation.

Accumulating studies have demonstrated that YAP activation is pivotal for the progression of many cancers.^34^ However, a comprehensive analysis of the TCGA database revealed that the expression level of YAP mRNA is not significantly correlated with overall survival in patients with various types of cancers, including HCC, colorectal carcinoma, lung carcinoma, and gastric carcinoma (data not shown). Similarly, our analysis of HCC tissue microarray (TMA) did not reveal any significant association between the protein level of YAP and overall survival of patients (supplementary Fig. 7a). TMA analysis revealed that the levels of YAP-R124me2a were significantly elevated in HCC- tissues compared to the surrounding noncancerous tissues (Fig. 5i). Furthermore, the increased levels of YAP-R124me2a were associated with higher malignancy grades of HCC (supplementary Fig. 7b). Importantly, high levels of YAP-R124me2a showed a significant correlation with poor prognosis in HCC patients (Fig. 5j). Similarly, the TMA analysis of colorectal carcinoma, lung carcinoma, and gastric carcinoma also revealed an inverse correlation between YAP-R124me2a levels in cancerous tissues and the overall survival of the patients (Fig. 5k; supplementary Fig. 7c–e). In contrast, the levels of total YAP protein were not associated with the prognosis of these patients (supplementary Fig. 7f–h). These findings suggest that YAP-R124me2a could serve as a potential biomarker and therapeutic target for multiple adenocarcinomas.

### A cell-permeable peptide disrupting the YAP-SOX9 interaction blocks YAP nuclear translocation and counteracts tumor progression

**Fig. 6:**
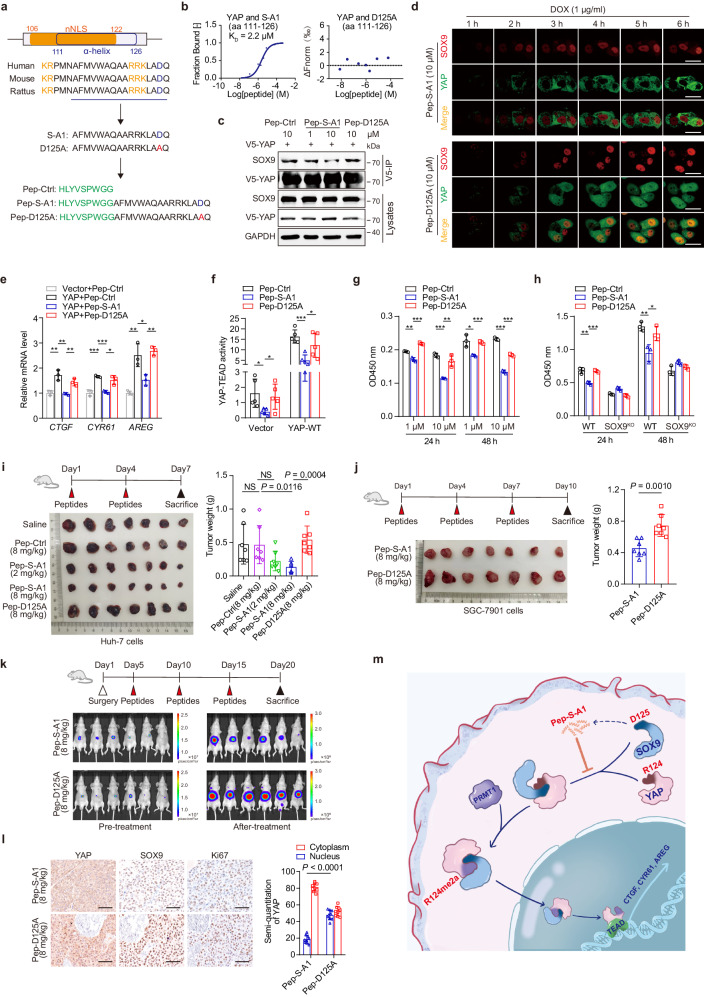
A cell-permeable peptide disrupting the YAP-SOX9 interaction attenuates tumor progression. **a** Identification of an α-helical peptide (S-A1) in the SOX9-HMG domain based on the YAP/SOX9-binding motif using the Glide program in Schrödinger. Cell-permeable peptides were generated by linking a cell-penetrating peptide to the α-helical peptide of SOX9. The peptide containing α-helices in SOX9 were predicted by the I-TASSER server (https://zhanggroup.org/I-TASSER/). **b** The binding affinities between YAP and the S-A1 (left) and D125A (right) peptides were determined by MST assay. **c** V5-IP was performed with the extracts of Huh-7 cells treated with different peptides at the indicated concentrations for 24 h and analyzed by immunoblotting with SOX9 antibody. **d** Live cell-imaging showed the SOX9-induced nuclear influx of YAP in Huh-7 cells treated with Pep-peptides. Scale bar = 20 μm. **e** qPCR analysis of *CTGF*, *CYR61* and *AREG* expression levels in Huh-7 cells treated with Pep-Ctrl, Pep-S-A1, or Pep-D125A (10 μM). Data are represented as mean ± SD, *P < 0.05, **P < 0.01, ***P < 0.001. **f** The 8×GTIIC reporter activity in Huh-7 cells treated with Pep-Ctrl, Pep-S-A1, or Pep-D125A (10 μM). Data are represented as mean ± SD, *P < 0.05, ***P < 0.001. **g** Pep-S-A1 inhibited the proliferation of Hep3B cells. Data are represented as mean ± SD, *P < 0.05, **P < 0.01, ***P < 0.001. **h** The proliferation of Huh-7 cells and SOX9^KO^ Huh-7 cells treated with Pep-peptides. Data are represented as mean ± SD, *P < 0.05, **P < 0.01, ***P < 0.001. **i** Schematic representation of the peptide therapy experiment. The Huh-7 xenografts were injected with different concentrat**i**ons of Pep-peptides at day 1 and day 4 (upper). Representative images of tumors derived from Huh-7 cell xenografts (lower). The weight of Huh-7 xenograft tumors (right). Data are represented as mean ± SD. **j** Schematic representation of the peptide therapy experiment for SGC-7901 xenograft tumors. Pep-peptides were injected into tumors at day 1, day 4, and day 7 after tumor formation (upper). Representative images of SGC-7901 cell xenografts (lower). The weight of SGC-7901 xenograft tumors (right). Data are represented as mean ± SD. **k** Schematic representation of the peptide therapy experiment using an orthotopic model of HCC (upper). Images of nude mice harboring Huh-7 cells stably expressing luciferase (lower). **l** Immunohistochemical staining of YAP, SOX9, and Ki67 in the tumor tissues described in K (left), Scale bar = 100 μm. Quantification of YAP staining in the cytoplasm and nucleus was performed using ImageJ software (right). **m** Schematic representation of the mechanism by which SOX9 triggers YAP nuclear entry via direct interaction

To confirm the role of the SOX9-YAP interaction in YAP activation and tumorigenesis, we used the I-TASSER server to predict potential α-helices in SOX9.^35^ We identified an α-helix within the HMG domain of SOX9 (amino acids 111–126), referred to as peptide S-A1. This helix contains D125, a key residue involved in the SOX9-YAP interaction (Fig. 6a). The MST assay confirmed that peptide S-A1 binds to YAP with a KD of 2.2 μM, and D125A mutation abolished this binding (Fig. 6b). To facilitate cellular uptake of the peptides, we generated two fusion peptides, Pep-S-A1 and Pep-D125A, by linking S-A1 and S-A1-D125A to a cell-penetrating peptide (Pep) via a di-glycine linker (Fig. 6a). Co-IP and PLA experiments showed that Pep-S-A1 disrupted the YAP-SOX9 interaction in HCC cells in a dose-dependent manner, while Pep-D125A had no effect (Fig. 6c; supplementary Fig. 8a, b). Treatment of Huh-7 cells with Pep-S-A1 reduced nuclear YAP levels, inhibited YAP-target gene expression and suppressed YAP-TEAD activity (Fig. 6d–f; supplementary Fig. 8c).

We then evaluated the antitumor effects of Pep-S-A1 both in vitro and in vivo. Pep-S-A1 inhibited HCC cell proliferation but had no effect on SOX9^KO^ Huh-7 cells (Fig. 6g, h). In a subcutaneous xenograft model of Huh-7 cells, Pep-S-A1 dose-dependently suppressed tumor growth, accompanied by reduced YAP nuclear staining and downstream gene transcription in the tumor tissues (Fig. 6i; supplementary Fig. 8d–f). Similar tumor growth inhibition was observed in SGC-7901 cell xenografts treated with Pep-S-A1 (Fig. 6j; supplementary Fig. 8g–i). We next utilized an orthotopic model of HCC to further confirm the antitumor effect of Pep-S-A1. Subcutaneous tumors established with luciferase-expressing Huh-7 cells were implanted into nude mouse liver. While there was no significant difference in luciferase activity of the tumors between the mouse groups before peptide administration (Fig. 6k; supplementary Fig. 8j), 2-week delivery of Pep-S-A1 significantly reduced the emitted bioluminescence and tumor growth compared with Pep-D125A, and notably inhibited YAP nuclear aggregation and downstream gene expression in the tumor tissues (Fig. 6k, l; supplementary Fig. 8j–l). Taken together, these data indicate that disruption of YAP-SOX9 interaction may be a promising strategy for tumor therapy (Fig. 6m).

## Discussion

The activation of mammalian YAP has been demonstrated to be crucial for multiple cancers. The upregulation of YAP and SOX9 has been observed across a range of tumor types, but the precise functional relationship between these two factors remains controversial in various tissues or cells.^15–18^ It has been reported that YAP transcriptionally upregulates the expression of SOX9 in hepatocyte-derived progenitor cells, indicating a potential synergistic relationship between them.^36^ Conversely, Liu et al. discovered that SOX9 is required for YAP-induced hepatocyte plasticity during hepatocarcinogenesis in mice. They further mentioned that SOX9 hampers the transcriptional activation of YAP by disrupting the DNA binding of YAP/TEAD in hepatocytes.^17^ However, in the present study, we found that SOX9 plays a crucial role in promoting YAP activation and enhancing its oncogenic properties during HCC progression. Importantly, we identified a direct interaction between YAP and SOX9 that mediates the nuclear translocation of YAP in HCC cells. Furthermore, our results indicate that disruption of the interaction of YAP and SOX9 effectively inhibits the growth of HCC. This finding suggests that SOX9 may play different roles in tumorigenesis and tumor progression of HCC.

Previous studies have indicated that the significance of NLS-containing proteins, such as ZO-2 and Mask1/2, in governing the nuclear translocation of YAP.^37,38^ This nuclear translocation process is fundamental to YAP’s oncogene function. A recent study has further illuminated this mechanism by demonstrating that the NLS of STAT3 is essential for YAP/TAZ nuclear translocation through an importin α3-mediated nuclear-shuttling machinery.^39^ Our current study reveals that SOX9-nNLS is required for YAP nuclear entry via CaM-mediated nuclear-shuttling machinery. These findings suggest a conventional mechanism of YAP nuclear entry via an NLS-containing shuttling partner. Moreover, our detailed analysis uncovered that Asp-125, a residue adjacent to the nNLS of SOX9, does not play a role in SOX9’s own nuclear translocation but is responsible for point-to-point binding between SOX9 and YAP and YAP nuclear entry. Thus, our study provides a novel framework to interpret the nuclear entry of YAP, i.e., SOX9 first interacts with YAP via direct binding between Asp-125 of SOX9 and Arg-124 of YAP and then directs YAP translocation into the nucleus via its nNLS. These findings unveil previously unidentified functions of SOX9 beyond its recognized role as a transcriptional factor.

Of note, the interest of this discovery is that the SOX9-binding fragment of YAP, the amino acid sequence spanning position 121 to 135, contains a 14-3-3 binding motif (RX1-2SX2-3S). Within this motif, residue R124 or S127 of YAP was predicted to simultaneously interact with the D125 residue of SOX9. However, we observed that mutating arginine 124 to lysine (R124K) blocked the interaction of YAP with SOX9, while mutating serine 127 to alanine (S127A) had no such effect. It was demonstrated previously that the phosphorylation of YAP at S127 leads to its sequestration in the cytoplasm by 14-3-3 proteins.^40^ Taken together, these data suggest that 14-3-3 binding motif of YAP, in which both R124 and S127 are located, interacts with distinct binding partners in different circumstances. The flexibility binding behavior might play a switch-like role in YAP nucleocytoplasmic translocation, a process that is crucial for controlling cell growth, proliferation, and differentiation.

YAP does not have a fixed three-dimensional structure in its unbound state,^41^ allowing for flexibility and adaptability. However, when it binds to its partners or undergoes modifications at the binding interface, YAP acquires a more defined structure, facilitating specific interactions.^42^ Arginine methylation disperses the charge over more atoms, and subsequently alters its hydrophobic properties, leading to a change in the binding of methylarginine to specific proteins and regulating their functions.^43^ Supporting this notion, we found that the asymmetric dimethylation at R124 of YAP (YAP-R124me2a), catalyzed by PRMT1, enhanced the YAP-SOX9 interaction. This strengthened interaction promotes the nuclear translocation of YAP, potentially influencing gene expression and cellular processes. We also observed a significant correlation between elevated levels of YAP-R124me2a, rather than total YAP, and a poorer prognosis among adenocarcinoma patients. These findings indicate that YAP-R124me2a might have the potential to be utilized as a biomarker and a therapeutic target for the treatment of various cancers.

The formation of YAP-partner complexes under certain pathological conditions makes YAP an ideal drug target.^44–46^ Dissociation of coactive YAP partners has been shown to have potential therapeutic effects in the treatment of cancers. For instance, verteporfin (a porphyrin compound) blocked the YAP-TEAD interaction and suppressed ovarian cancer growth in mice.^47^ Similarly, a rationally engineered ‘super-TDU’ peptide derived from VGLL4, which specifically disrupts the YAP-TEAD interaction, has been shown to effectively halt the progression of gastric cancer.^48^ In light of this, impeding YAP nuclear localization directed by SOX9 emerges as another strategy to inhibit YAP activity and subsequently suppress tumor growth. It has been reported that α-helical structures play a pivotal role in mediating many protein-protein interactions (PPIs), and they represent a generic template for the design of PPI inhibitors.^49^ Indeed, we developed a peptide-mimicking α-helix derived from the SOX9 fragment, designated as Pep-S-A1. This peptide effectively disrupts the SOX9-YAP interaction, preventing the nuclear localization and oncogenic function of YAP, and exhibiting significant therapeutic effects in mouse tumor models. However, for the applicability of these findings, future research should be taken, including the specificity and safety of peptide-based drugs. Overall, these data not only further confirm the nuclear import of YAP via its direct interaction with SOX9 but also provide compelling evidence that disruption of SOX9-partner interactions represents a valuable approach with potential application for cancer therapy. The development of agents like Pep-S-A1 paves the way for targeted therapeutics aimed at disrupting oncogenic PPIs, ultimately leading to more effective cancer treatments.

In summary, our study unveils a previously unrecognized function of SOX9 as a ferry regulator of a critical oncogene, extending its role beyond its intrinsic role as a transcription factor. We also elucidate a novel molecular mechanism that underlies YAP nuclear transportation. Our findings also demonstrate that PRMT1-catalyzed YAP-R124me2a promotes YAP translocation and may serve as a general biomarker for predicting survival outcomes in cancer patients. More interestingly, we demonstrate that dissociation of the SOX9-YAP complex is a potential approach to treat YAP-driven human cancers.

## Materials and methods

### Ethics approval and consent to participate

All patients whose specimens were utilized in this study have signed the informed consent form granting permission for the use of their specimens in scientific research. All animal experiments in the current study were performed in accordance with the National Institutes of Health Guide for the Care and Use of Laboratory Animals and were approved by the Scientific Investigation Board of Naval Medical University.

### Generation of knockout cell lines with CRISPR-Cas9

SOX9 knockout cells were created using the CRISPR-Cas9 system at the Cyagen Company. Huh-7 or 293 A cells were transiently transfected with a plasmid carrying the guide DNA for SOX9 and then were seeded into a 96-well plate to form monoclines. Knockout clones were screened by PCR and verified by Sanger sequencing. The guide sequence for human SOX9 was GCTCGGACACCGAGAACACG-CGG.

### RNA sequencing

The total RNA of Huh-7 and SOX9^KO^ Huh-7 cells was extracted using TRIzol Reagent (Life Technologies, 15596026). The RNA quality (RNA integrity number, RIN) was checked with an Agilent Bioanalyzer, and confirmed a high integrity RIN > 7 for all samples. RNA sequencing libraries were prepared with the VAHTS Universal V6 RNA-seq Library Prep Kit (Vazyme NR604-02) according to the provided instructions. Sequencing was performed in the single read mode using an Illumina Novaseq 6000. Sequenced reads were aligned to the GRCh38 reference genome with TopHat2 (v 2.0.14). RNA sequencing data can be accessed publicly on GEO under the accession number GSE214335.

### Human Tumor TMA

Paired cancer and adjacent non-cancer paraffin tissue sections of human hepatocellular carcinoma (LIHC), lung adenocarcinoma (LUAD), colorectal adenocarcinoma (COAD), and gastric adenocarcinoma (STAD) with detailed pathological information were purchased from Shanghai Core Bio-Tech Co., Ltd. (chip number: HLivH180Su11, HlivH180Su16 [a copied version of HlivH180Su11], and HLivH180Su15 for LIHC; HLivH180Su11 for the YAP and R124me2a experiment in Fig. 5 and supplementary Fig. 7; HlivH180Su11/16 and HlivH180Su15 for the analysis of YAP and SOX9 nuclear levels; HLugA180Su07 for LUAD; HColA180Su17 for COAD; and HStmA180Su19 for STAD). Immunohistochemistry staining was measured by independent pathologists. The staining intensity score ranged from 0 to 3, reflecting the percentage of immunoreactive staining area in the sample (0–10% scored as 0, 11–25% as 1, 26–75% as 2, and 76–100% as 3). The final total score for each specimen was determined by multiplying the staining intensity score with the staining extent score. For the correlation analysis of nuclear levels of SOX9 and YAP in TMAs, three fields with different staining intensities in each specimen were selected to measure the positive staining location, and the average values of the three fields were taken as positive rates by independent pathologists. The tissue sample in which the YAP or SOX9 nuclear expression level is zero was not statistical.

### Live cell imaging

Huh-7 cells were infected with lenti-YAP-linker-EGFP-rtTA3 followed by infection with lenti-SOX9-linker-mCherry-rtTA3, with selection using puromycin (Sigma-Aldrich, P9620) or Blasticidin S hydrochloride (Yeasen,60218ES10), and then plated on confocal 6-well plates (Biosharp, BS-15-GJM) at a suitable concentration. Approximately 1 h after doxycycline (DOX, Yeasen, 60204ES03) treatment, live cell images were captured every 30 min using an Olympus SpinSR10 microscope at ×200 magnification. For the PLA, the pretreated cell samples were fixed after DOX induction for 12 h. DOX was administered to the infected Huh-7 cells at a concentration of 1 μg/mL.

### Luciferase reporter gene assay

Huh-7 cells were transfected with a vector expressing full-length or truncated SOX9 together with a TEAD4-related reporter plasmid, 8×GTIIC (RIKEN, Japan). The pRL-SV40 (Promega, E2261) vector was transfected into all the treated cells and was used to normalize the data. Luciferase reporter gene assays were assayed with the Dual-Luciferase Reporter Assay System (Promega, E2920). All experiments were replicated a minimum of three times.

### Cytoplasmic and nuclear fractionation

Cytoplasmic and nuclear fractionation was accessed by the Minute^TM^ Cytoplasmic and Nuclear Fractionation kit (Invent, SC-003) following the manufacturer’s recommendations with some modifications made by us to optimize the protocol. Cells were resuspended in pre-chilled cytoplasm extraction buffer and incubated on ice for 5 min. After a thorough one-minute vortex, the supernatant (cytoplasmic proteins) was obtained by centrifuging at 12,000 rpm for 5 min. To eliminate cytoplasmic proteins, the pellet was washed with pre-chilled PBS, and subsequently resuspended in a nuclear extraction buffer. The sample was vortexed thoroughly for 3 min in an ice bath, and these steps were repeated four times. The precipitate was discarded, and the supernatant (nuclear proteins) was saved for further analysis.

### The protein pull-down assay

FLAG-YAP protein (20 μM) purified from SF9 was incubated with 20 μM GST-SOX9 protein (Abcam, ab131911), GST protein, or blank control in the presence of FLAG beads (Sigma-Aldrich, A2220) overnight at 4 °C. Following this, the beads were washed two times in binding and wash buffer (20 mM Tris [pH 8.0], 150 mM NaCl, 0.5% NP40, 0.5% TritonX-100, 1 mM DTT, and 10% glycerol), and one wash with high salt buffer (20 mM Tris [pH 8.0], 250 mM NaCl, 0.5% NP40, 0.5% TritonX-100, 1 mM DTT, and 10% glycerol). The slurry was boiled with SDS buffer and then subjected to immunoblot.

### Microscale thermophoresis

His-tagged YAP and the SOX9-HMG domain purified from *Escherichia coli* were labeled with 25 nM RED-tris-NTA 2nd Generation dye at room temperature for 10 min. Then, the labeled protein was incubated with a serially diluted peptide or protein for 10 min at room temperature. MST buffer containing 50 mM Tris-HCl [pH 7.4], 150 mM NaCl, and 0.05% Tween-20 was used in all measurements. The solution was centrifuged for 10 min at 4 °C and 15,000 g, and the supernatant was transferred to a fresh tube. The sample was loaded into the capillaries and measured at 40% LED/excitation power and medium MST power. The *K*_D_ was determined in MO. Affinity Analysis using the *K*_D_ fit.

### DUOlink proximity ligation assay (PLA)

The pretreated cell samples were grown on coverslips, fixed in 4% paraformaldehyde (PFA) for 15 minutes, and then washed two times in PBS. Following a 1-hour incubation at 37 °C with blocking solution® (Sigma-Aldrich, DUO82007), the cells were incubated with primary antibodies at optimal concentrations overnight at 4 °C. After three washes in Buffer A® (Sigma-Aldrich, DUO82049), the samples were incubated with a 1:5 dilution of PLUS® and MINUS® Duolink In Situ PLA Probe (Sigma-Aldrich, DUO92002 and DUO92004) for 1 hour at 37 °C. Three additional washes in Buffer A® preceded a 30-minute incubation with 1 unit/μL of T4 DNA ligase diluted in ligase buffer (1:5, Sigma-Aldrich, DUO92013) at 37 °C. Subsequently, cells were washed three times again in Buffer A® and treated with 5 units/μL of DNA polymerase in diluted polymerase buffer (1:5, Sigma-Aldrich, DUO92013) for 100 min at 37 °C. Finally, cells were washed twice for 10 minutes each in 1× Buffer B® (Sigma-Aldrich, DUO82049), then quickly washed for 1 min in 0.01× Buffer B®. The slides were mounted using DUOlink in situ mounting medium containing DAPI, and the images were captured using a Leica TCS SP8 confocal microscope.

### Molecular docking

Prediction of the protein-peptide complex was performed using the Glide program in Schrödinger. The SOX9-HMG domain crystal structure (PDB code 4EUW) was downloaded as a docking model and optimized using the Protein Preparation Wizard procedure, then a receptor grid file was generated using the Receptor Grid Generation procedure. The predicted structure of YAP was downloaded from the AlphaFold Protein Structure Database (AF-P46937-F1), and residues 121–135 of this structure were extracted in PyMOL. The Ligandpre procedure was used to generate the protonation state and stereoisomer for the YAP peptide. Finally, the standard precision (SP-Peptide) mode in Ligand Docking was chosen to complete the molecular docking. Visualization of the interaction was performed in PyMOL.

### Mass spectrometry

Huh-7 cells were first infected with PRMT1 lentivirus for 48 h, and then transfected with V5-tagged YAP for an additional 24 hour, solubilized in IP buffer (Pierce), and immunoprecipitated with V5 beads (Sigma-Aldrich, A7345) overnight at 4 °C. The immunoprecipitants were washed three times with wash buffer (20 mM Tris [pH 8.0], 150 mM NaCl, 0.5% NP40, 0.5% TritonX-100, and 10% glycerol) and washed two times with PBS buffer. The proteins attached to the beads were subjected to trypsin digestion in a suitable buffer. Tryptic peptides were analyzed by mass spectrometry on a Q ExactiveTM HF-X mass spectrometer (Thermo Scientific).

### YAP Arg-124-methylation antibody

The polyclonal antibodies specific to Arg-124 (R124)-methylated YAP were generated in rabbits by Chinapeptides Co., Ltd (Shanghai, China) using the methylated-peptide CTPQHVR(me)AHSSP-NH2, CTPQHVR(me2a)AHSSP-NH2, or CTPQHVR(me2s)AHSSP-NH2 and affinity-purified.

### Dot blot analysis

The peptides used for the dot blot assay were synthesized at ChinaPeptides, and the sequences are provided in supplementary Table 1. Prior to the assay, peptides were diluted to 10 μM using sterile ddH_2_O, and 1 μL was spotted onto a nitrocellulose membrane (PALL, HAHY00010). The membranes were dried at room temperature and then blocked with 5% skimmed milk. Subsequently, the membranes were incubated with the antibodies, following a similar protocol as that used in immunoblotting analysis.

### In vitro methyltransferase assay

PRMT1 protein was overexpressed in 293 A cells using lenti-PRMT1 and immunoprecipitated using anti-FLAG beads. The washed immunocomplexes were incubated at 30 °C for 90 min in the presence of 25 μM YAP protein purified from *E. coli* and 250 μM S-adenosylmethionine (SAM, Sigma-Aldrich, A4377) with 20 μL reaction buffer (20 mM Tris [pH 8.0], 50 mM KCL,1 mM DTT, 1 mM PMSF). After the methylation reaction, the sample was boiled for 5 min, and then the PRMT1 and the methylation level of YAP were detected by immunoblot.

### Generation of cell-penetrating peptides

Peptides containing an α-helix corresponding to the SOX9-HMG domain fused with a cell-penetrating peptide (Pep, HLYVSPW) at the N terminus were synthesized at Chinapeptides Co., Ltd (Shanghai). Sequences of peptides used in this study are listed in supplementary Table 1.

### Statistical analysis

All data were analyzed by Prism 7.0 (GraphPad), and data are representative of at least three biologically independent experiments. Two-group datasets were analyzed by Student’s unpaired *t* test. Linear regression analysis was performed with the GraphPad Prism 7.0 software *χ*^2^ test. Kaplan–Meier analysis and log-rank tests were performed to compare the survival probabilities between different groups. Statistical tests were two-tailed and a *P* value less than 0.05 was considered statistically significant. For all figures unless otherwise noted, * indicates *P* < 0.05, ** indicates *P* < 0.01, *** indicates *P* < 0.001, NS indicates no significant difference.

### Supplementary information

supplementary materials

original western blot figures

Supplementary Table 1

Supplementary Table 2

Supplementary Table 3

## Data Availability

All data to support the conclusions in this manuscript can be found in the main text or the supplemental materials. Genomic data are publicly available at the GEO (accession number GSE214335). Raw data from our study may be requested from the corresponding authors.

## Materials availability

All stable and unique reagents generated in this study are available upon reasonable request.
