# Supplementary material for: SRY-Box transcription factor 9 triggers YAP nuclear entry via direct interaction in tumors
Source: Signal Transduct Target Ther. 2024 Apr 24;9:96. doi: 10.1038/s41392-024-01805-4 (PMC11039692; doi:10.1038/s41392-024-01805-4)
Supplement: Supplementary file 1 — supplementary materials [file 41392_2024_1805_MOESM1_ESM.docx]

Supplementary Materials for

SRY-Box transcription factor 9 triggers YAP nuclear entry via direct interaction in tumors

Hui Qian, Chen-Hong Ding, Fang Liu, Shi-Jie Chen, Chen-Kai Huang, Meng-Chao Xiao, Xia-Lu Hong, Ming-Chen Wang, Fang-Zhi Yan, Kai Ding, Ya-Lu Cui, Bai-Nan Zheng, Jin Ding, Cheng Luo^✉^, Xin Zhang^✉^ and Wei-Fen Xie^✉^

Correspondence to: [cluo@simm.ac.cn](mailto:cluo@simm.ac.cn); [zhang68@hotmail.com](mailto:zhang68@hotmail.com);

[weifenxie@medmail.com.cn](mailto:weifenxie@medmail.com.cn)

**This PDF file includes:**

Materials and methods

Supplementary figures 1-8.

**Materials and methods**

**Human tissue samples**

The specimens were collected from patients who received surgical resection and were diagnosed by dedicated pathologists at the Eastern Hepatobiliary Surgery Hospital (Shanghai, China).

**Cell lines and culture conditions**

Huh-7, PLC, HEK-293A/293T, HCT-116 and A549 cells were obtained from American Type Culture Collection. SGC-7901 was obtained from the National Collection of Authenticated Cell Cultures (Shanghai). Cell lines were cultured at 37°C in a 5% CO2 atmosphere and were routinely tested for mycoplasma contamination using a Mycoalert detection kit (Lonza, LT07-318) and authenticated by short tandem repeat analysis. Huh-7, PLC, HEK-293A/293T, SGC-7901 and A549 cells were cultured in Dulbecco's Modified Eagle Medium (Gibco, 11995073). HCT-116 cells were cultured in McCoy’s 5A (Gibco, 16600108). All media were supplemented with 10% FBS (GIBCO, 10099141C).

**Mice**

Yapflox/flox mice were a gift from Professor Duojia Pan at Howard Hughes Medical Institute. All mice were born and maintained under pathogen-free conditions.

**Adeno-associated viruses**

The coding sequences of wild-type and mutant YAP were inserted into pENN-AVV-TBG-PI-RBG (Penn Vector Core). Replication-incompetent AAV2/8 viruses expressing YAP and its mutant variants under the control of the TBG promoter were packaged and purified by Vigene Bioscience (Jinan, China).

**Gene overexpression, silencing, and transfection**

The full-length cDNAs, truncated cDNAs, and mutated cDNAs were synthesized by The Beijing Genomics Institute (Beijing, China) and subcloned into vectors according to standard molecular cloning methods. Gene-specific small interfering RNAs (siRNAs) were obtained from GenePharma (Suzhou, China). Transient transfection of expression vectors or siRNA using Lipofectamine^TM^ 2000 (Thermo Fisher Scientific, 11668019) was performed following the manufacturer's protocol. Final concentrations for all siRNAs were 20 nmol/L. Sequences of siRNAs used in this study are listed in supplementary Table 2.

**Lentivirus**

The coding sequence of wild-type or mutated YAP and SOX9 and wild-type PRMT1 were subcloned into pCDH-CMV-MCS-EF1-copGFP (System Biosciences) in frame with the indicated tags. Lentivirus was produced by co-transfecting 293T cells with the expression vector together with packaging plasmid psPAX2 (Addgene, 12260) and envelope plasmid pMD2.G (Addgene, 12259) using Lipofectamine^TM^ 2000 (Thermo Fisher Scientific, 11668019) according to the manufacturer’s instructions. Viral supernatant was harvested at 48–72 h post-transfection. The supernatant was centrifuged at 3,000 rpm for 5 min at 4°C to remove cell debris and then was passed through a 0.45 μm MF-Millipore® Membrane filter (Sigma-Aldrich, HAWP04700). Filtered viruses were used directly or stored in cryovials at -80°C until use.

**Total RNA isolation and real-time PCR**

Total RNA was isolated from cells or tissues with RNAiso Plus Reagent (TaKaRa, 9108). SuperScript Ⅲ reverse transcriptase (Invitrogen,18080093) was employed to synthesize first-strand cDNA. Real-time PCR was performed in an ABI Step One Real-time Detection System (Life Technologies) using SYBR Green (Takara, RR420A). The primer sequences are listed in supplementary Table 2.

**Protein preparation, co-immunoprecipitation, and immunoblotting**

For protein preparation, cells were lysed in 2× sodium dodecyl sulfate (SDS) protein lysis buffer (4% SDS, 20% glycerol, 100 mM Tris-Cl [pH 6.8], with PMSF or other protease inhibitors). For co-immunoprecipitation (Co-IP), samples were prepared using Pierce^TM^ IP lysis buffer (Thermo Fisher Scientific, 87787) following the manufacturer's recommendations. Lysates were centrifuged at 12,000 rpm for 10 min, and the supernatants were collected. The protein concentration was determined using a BCA kit (Beyotime, P0010, Shanghai, China). For Co-IP samples, 1 mg of protein was precipitated with 20 μL of agarose-conjugated antibodies. For immunoblotting, protein samples were separated using SDS-polyacrylamide gel electrophoresis and then transferred onto nitrocellulose membranes (Millipore, HAHY00010).

The membranes were blocked with 5% milk or bovine serum albumin in 1‰ PBS-T buffer, then incubated with primary antibodies overnight at 4°C. The secondary antibodies also were diluted appropriately using milk or bovine serum albumin in PBS-T (IRDye 680, LiCor Biosciences, 929-70050, and IRDye 800, LiCor Biosciences,929-70020). The antibodies are listed in supplementary Table 3.

**Immunofluorescence staining**

For cell immunofluorescence staining, the following procedures were used. Cells were grown on coverslips, initially washed with PBS, then fixed in 4% PFA, and permeabilized with 0.3% Triton X-100 diluted with PBS for 5 min at room temperature. Slides were incubated in blocking buffer (5% normal donkey serum diluted with PBS). Primary antibodies were incubated at 4°C overnight, followed by incubation with Alexa Fluor 488- and Alexa Fluor 546-conjugated secondary antibodies (Thermo Fisher, Waltham, MA, USA). The slides were mounted with DAPI-containing medium (Sigma-Aldrich, DUO82040), and the images were acquired with a Leica TCS SP5 confocal microscope.

**Immunohistochemistry**

Formaldehyde-fixed, paraffin-embedded sections of liver or tumor tissues were used for histopathological examination. Standard staining with hematoxylin and eosin or immunohistochemistry analysis was performed on sections 4 mm in thickness from each specimen block and observed using a photomicroscope (Olympus, Tokyo, Japan).

**Human HCC xenograft model**

Huh-7 cells (1×106) or Huh-7 cells infected with different viruses were injected subcutaneously into the right flank of male athymic BALB/c nude mice (5 weeks old). Tumor volumes were measured and analyzed using the equation: volume = π/6×length×(width)2.Once the average tumor volume reached approximately 60 mm3, the mice were randomly divided into five groups (eight mice per group) and treated with intratumorally injections of Pep-S-A1, Pep-D125A, or saline twice weekly. For the orthotopic HCC model, 1×106 Huh-7 cells expressing luciferase were injected subcutaneously into the right flank of BALB/c nude mice to establish tumor xenografts. The tumor nodules derived from xenograft model and then were divided into 1 mm3 pieces and subsequently implanted into the left lobe of the livers of 5-week-old male BALB/c nude mice to mimic primary HCC. The mice were randomly separated into two groups (eight animals per group) according to the bioluminescence 3 days after implantation. Pep-S-A1 or Pep-D125A was then injected via the tail vein every 5 days. The mice were regularly monitored using a luminal imaging system (Caliper) once each week and killed 2 weeks post tumor fragments transplanted.

Supplementary figures


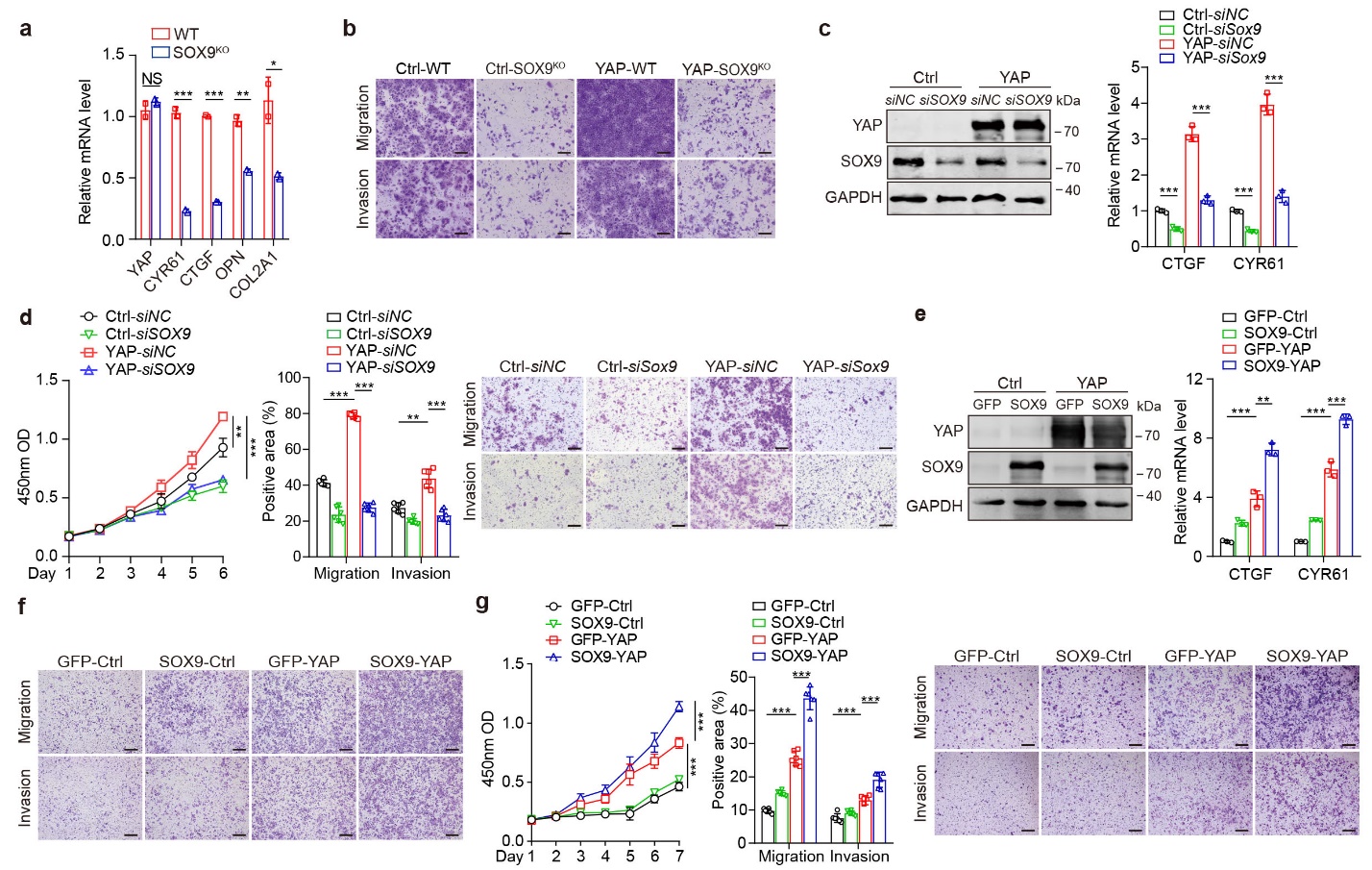


**Supplementary Fig. 1 SOX9 is essential for YAP activation in HCC cells.** **a** qPCR analysis of *YAP, CYR61, CTGF, OPN* and *COL2A1* in Huh-7 cells and SOX9^KO^ Huh-7 cells. Data are represented as mean ± SD. **P* < 0.05, ***P* < 0.01, ****P* < 0.001, NS, not significant. **b** Representative images of the migration and invasion of Huh-7 cells and SOX9^KO^ Huh-7 cells infected with lenti-YAP and control virus. Scale bar = 100 μm. **c** PLC cells infected with lenti-YAP or control virus (Ctrl) were transfected with *siSOX9* or *siNC*, and the YAP and SOX9 expression levels were detected by immunoblotting (left); the *CTGF* and *CYR61* mRNA levels were examined (right), data are represented as mean ± SD, ****P* < 0.001. **d** The proliferation capacity (left), and migration and invasion capacity of the cells described in c were measured (middle), data are represented as mean ± SD, ***P* < 0.01, ****P* < 0.001. Representative images of the migration and invasion of PLC cells in c (right). Scale bar = 100 μm. **e** YAP and SOX9 were upregulated in PLC cells by lentivirus and adenovirus, and the YAP and SOX9 expression levels were detected by immunoblotting (left); qPCR analysis of *CTGF* and *CYR61* in PLC cells (right), data are represented as mean ± SD, ***P* < 0.01, ****P* < 0.001. **f** Representative images of the migration and invasion of Huh-7 cells in Fig 1g. Scale bar = 100 μm. **g** The proliferation capacity (left), and migration and invasion capacity of the cells described in f were measured (middle), data are represented as mean ± SD, ****P* < 0.001. Representative images of the migration and invasion of PLC cells in e (right). Scale bar = 100 μm.


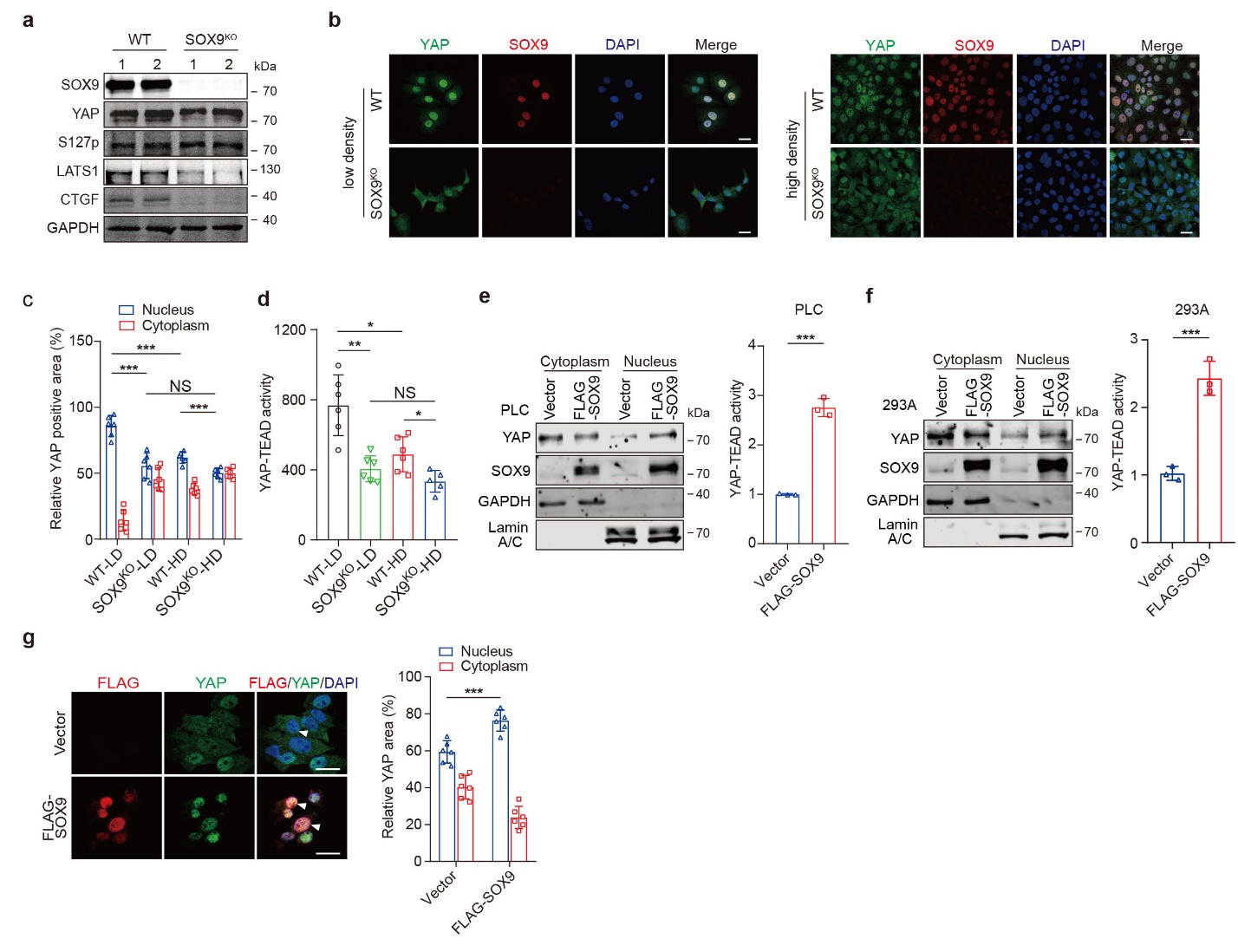


**Supplementary Fig. 2 SOX9 promotes YAP nuclear translocation. a** Immunoblot analysis of SOX9, YAP, phosphorylated YAP (S127p), LATS1, and CTGF protein levels in Huh-7 cells and SOX9^KO^ Huh-7 cells. GAPDH was used as the control. **b** Immunofluorescence staining with YAP and SOX9 antibodies in Huh-7 cells at different cell densities, scale bar = 20 μm. **c** The quantification of green staining in the cytoplasm and nucleus was performed using ImageJ software. Data are represented as mean ± SD, NS, not significant, ****P* < 0.001. **d** The 8×GTⅡC reporter activity in Huh-7 cells at different cell densities. Data are represented as mean ± SD, NS, not significant, **P* < 0.05, ***P* < 0.01. **e** Immunoblot analysis of YAP in the cytoplasmic and nuclear fractions of PLC cells transfected with control (Vector) or FLAG-SOX9 plasmid (left). The 8×GTⅡC reporter activity in PLC cells transfected with control (Vector) or SOX9 plasmid (right). Data are represented as mean ± SD, ****P* < 0.001. **f** Immunoblot analysis of YAP in the cytoplasmic and nuclear fractions of 293A cells transfected with control (Vector) or FLAG-SOX9 plasmid (left). The 8×GTⅡC reporter activity in 293A cells transfected with control (Vector) or SOX9 plasmid (right). Data are represented as mean ± SD, ****P* < 0.001. **g** Immunofluorescence staining with FLAG and YAP antibodies in Huh-7 cells transfected with FLAG-SOX9 or control (Vector) plasmid (left). Scale bar = 20 μm. The quantification of green staining in the cytoplasm and nucleus was performed using ImageJ software (right). Data are represented as mean ± SD, ****P* < 0.001.


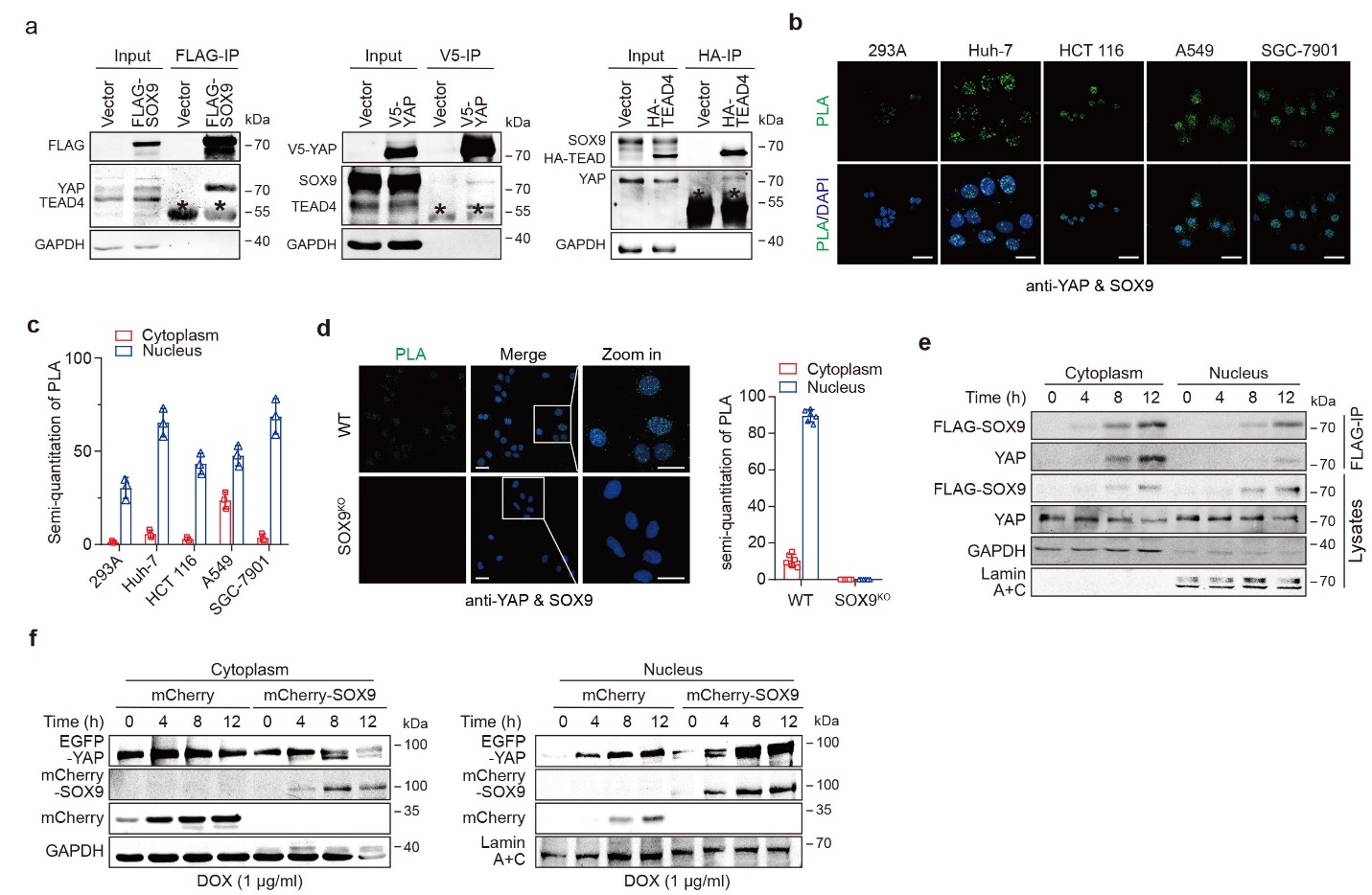


**Supplementary Fig. 3 The interaction between SOX9 and YAP mediates YAP nuclear translocation. a** Co-immunoprecipitation was performed with extracts from Huh-7 cells transfected with FLAG-SOX9 using anti-FLAG beads (left), from cells transfected with V5-YAP using anti-V5 beads (middle) and from cells transfected with HA-TEAD4 using HA antibody (right), and proteins were detected by immunoblotting. * indicates heavy chain. **b** Representative images of PLAs using antibodies against SOX9 and YAP in different cells. Green spots indicate a YAP/SOX9 protein interaction, and DAPI-stained nuclei are blue. Scale bar = 20 μm. **c** The green spots in the cytoplasm and nucleus were quantified using ImageJ software. **d** Representative images of PLAs using antibodies against SOX9 and YAP in Huh-7 and SOX9^KO^ Huh-7 cells (left). The green spots in the cytoplasm and nucleus were quantified using ImageJ software (right). Scale bar = 20 μm. **e** Immunoprecipitation was performed with cytoplasmic and nuclear extracts from Huh-7 cells transfected with FLAG-SOX9 at different times using anti-FLAG beads. **f** Immunoblot analysis of YAP in the cytoplasmic and nuclear fractions of Huh-7 cells with DOX-inducible co-expression of EGFP-YAP and mCherry-SOX9 after treatment with DOX for the indicated times.


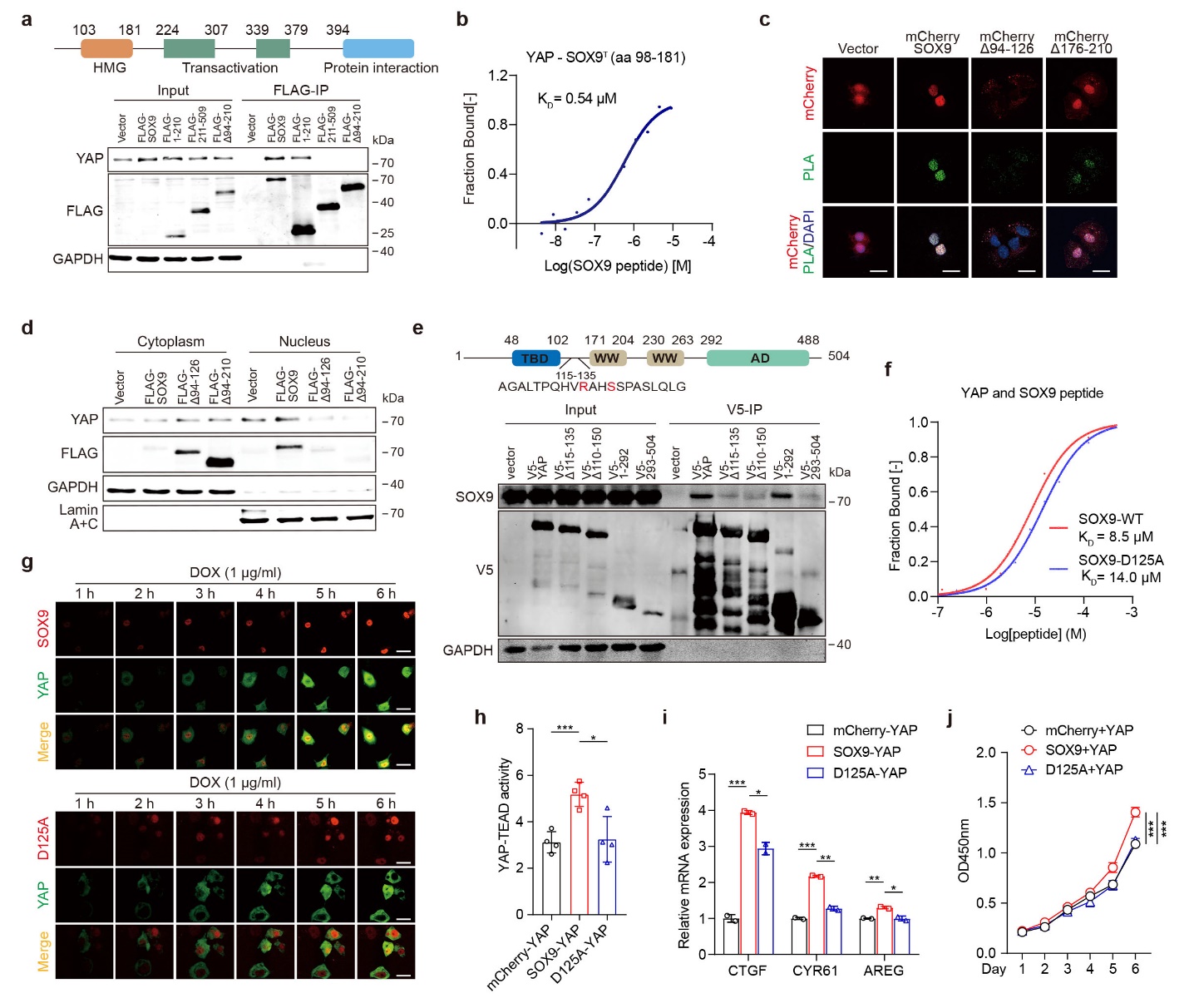


**Supplementary Fig. 4 YAP activation depends on the interaction of the Asp-125 residue of SOX9 with YAP. a** Illustration of SOX9 protein domains (upper). Huh-7 cells were transfected with FLAG-tagged SOX9 variants (1–210, 211–509, Δ94–210). The regions of SOX9 interacting with YAP were analyzed by immunoprecipitation with anti-FLAG beads followed by immunoblot detection (lower). **b** MST assay to determine the binding affinity between the YAP protein and HMG domain of SOX9 (aa 98–181). **c** Representative images of PLA with antibodies against mCherry and YAP in Huh-7 cells with DOX-inducible expression of mCherry fusion protein after treatment with DOX for 24 h. Scale bar = 20 μm. **d** Immunoblot analysis of YAP in the cytoplasmic and nuclear fractions of Huh-7 cells transfected with full-length FLAG-SOX9 or its deletion mutants (Δ94–126, Δ94–210). **e** Illustration of YAP protein variants used in binding assays (upper). Immunoprecipitation was performed with the cell extracts from Huh-7 cells transfected with V5-tagged YAP variants using anti-V5 beads, and the binding of SOX9 was examined by immunoblot analysis (lower). **f** MST assay to determine the binding affinity between YAP protein and wild-type (WT) peptide or D125A peptide. **g** Live cell imaging showing YAP nuclear influx in Huh-7 cells with DOX-inducible co-expression of EGFP and mCherry fusion proteins. Scale bar = 20 μm. **h** The 8×GTⅡC reporter activity in Hep3B cells with DOX-inducible EGFP-YAP and mCherry fusion proteins. Data are represented as mean ± SD, **P* < 0.05, ****P* < 0.001. **i** qPCR analysis of *CTGF, CYR61* and *AREG* expression in Hep3B cells with DOX-inducible EGFP-YAP and mCherry fusion proteins. Data are represented as mean ± SD, **P* < 0.05, ***P* < 0.01, ****P* < 0.001. **j** The proliferation of Hep3B cells with DOX-inducible co-expression of EGFP-YAP and mCherry fusion proteins. Data are represented as mean ± SD, ****P* < 0.001.


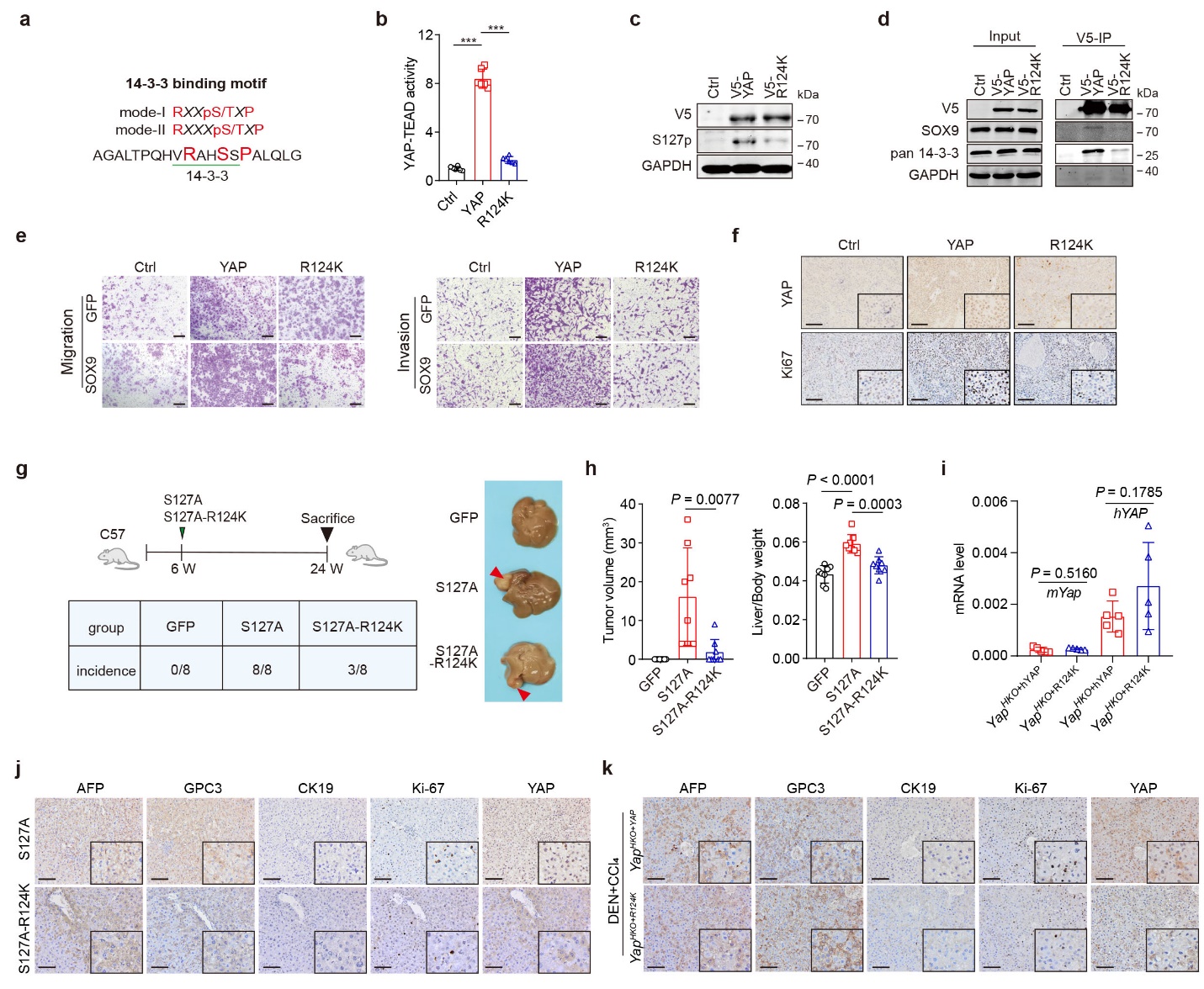


**Supplementary Fig. 5 The Arg-124 residue of YAP affects the YAP malignancy function in HCC. a** The major consensus binding motifs of 14-3-3 and the consensus sequence of human YAP around residues R124 and S127. **b** The 8×GTⅡC reporter activity in Huh-7 cells infected with lenti-YAP or lenti-R124K. Data are represented as mean ± SD, ****P* < 0.001. **c** Immunoblot analysis of YAP and phosphorylated YAP in Huh-7 cells infected with lenti-YAP or lenti-R124K. **d** Co-immunoprecipitation was performed using anti-V5 beads in Huh-7 cells infected with lenti-YAP or lenti-R124K. The interaction of SOX9 or 14-3-3 with YAP was further analyzed by immunoblot analysis. **e** Huh-7 cells were infected with lenti-YAP, lenti-R124K, or control (Ctrl) virus and then infected with Ad-SOX9 or Ad-GFP. Representative images of migration (left) and invasion (right) in Huh-7 cells. Scale bar = 100 μm **f** Immunohistochemical staining for YAP and Ki67 in Huh-7 xenografts, Scale bar = 100 μm (right). **g** Schematic representation of an active YAP^S127A^-induced hepatocarcinogenesis mouse model (upper). Six-week-old mice were co-injected with AAV-TBG-Cre and AAV-TBG-YAP^S127A^ or AAV-TBG-YAP^S127A-R124K^. The incidence of liver tumors was scored at 18 weeks after the injection of the AAV virus (lower). n = 8 in each group. Representative images of the livers with tumors (right). **h** The tumor volume (left) and liver/body weight ratio (right) of the mice described in g. **i** The mRNA levels of mouse *Yap* (*mYap*) and human *YAP* (*hYAP*) in liver tissue isolated from a DEN plus CCl_4_-induced HCC model. **j** Immunohistochemical staining of AFP, GPC3, CK-19, Ki67 and YAP in liver tissue isolated from a HCC model treated with YAP-S127A. Scale bar = 100 μm. **k** Immunohistochemical staining of AFP, GPC3, CK-19, Ki67 and YAP in liver tissue isolated from a DEN plus CCl_4_-induced HCC model. Scale bar = 100 μm.


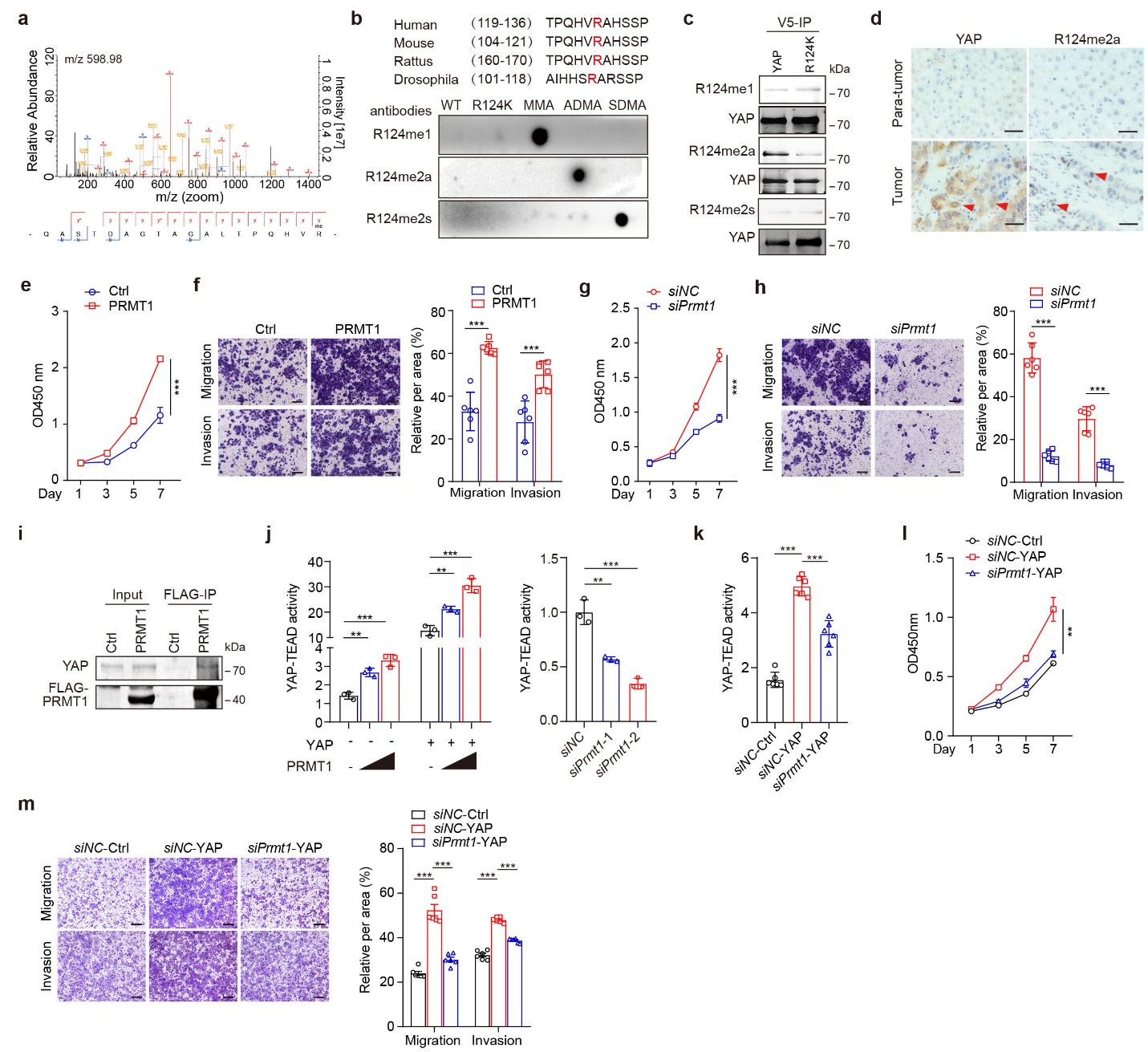


**Supplementary Fig. 6** **PRMT1 mediated the asymmetric dimethylation of YAP at R124. a** Mass spectrometry analysis of a YAP fragment (aa 116–124) precursor ion (m/z = 598.98) revealed methylation of YAP at arginine 124. **b** Alignment of YAP methylation motif sequences from different organisms (upper). Dot blot assays were performed with the peptide (119–136) of YAP (WT) or with mono-methylarginine modification (MMA), asymmetric dimethylarginine (ADMA), or symmetric dimethylarginine (SDMA), or mutation at R124 residue (R124K) to determine the specificity of antibodies recognizing different methylation modifications of YAP (lower). **c** Immunoblot analysis of YAP and methylated YAP in Huh-7 cells infected with lenti-YAP and lenti-R124K. **d** Immunohistochemical staining of YAP and R124me2a in human HCC tissue and paired para noncancerous tissue, scale bar = 100 μm. **e-f** The proliferation capacity (**e**), and migration and invasion capacity (**f**) of the Huh-7 cells transfected with control (Ctrl) or Lenti-PRMT1 (PRMT1), data are represented as mean ± SD. ****P* < 0.001. Scale bar = 100 μm. **g-h** The proliferation capacity (**g**), and migration and invasion capacity (**h**) of the Huh-7 cells transfected with *siNC* or siRNA against *Prmt1* (*siPrmt1*). data are represented as mean ± SD. ****P* < 0.001. Scale bar = 100 μm. **i** The interaction between PRMT1 and YAP was detected by co-immunoprecipitation (FLAG-IP). **j** The dose-dependent 8×GTⅡC reporter activity in Huh-7 cells infected with lenti-PRMT1 and then transfected with YAP or control plasmid (left), and the 8×GTⅡC reporter activity in Huh-7 cells transfected with *siPrmt1* or *siNC (*right). Data are represented as mean ± SD, ***P* < 0.01, ****P* < 0.001. **k-m** The 8×GTⅡC reporter activity **(k)**, the proliferation capacity (**l**), and migration and invasion capacity (**m**) was detected in the Huh-7 cells treated with Lenti-YAP and *siPrmt1* simultaneously. data are represented as mean ± SD. ***P* < 0.01, ****P* < 0.001. Scale bar = 100 μm.


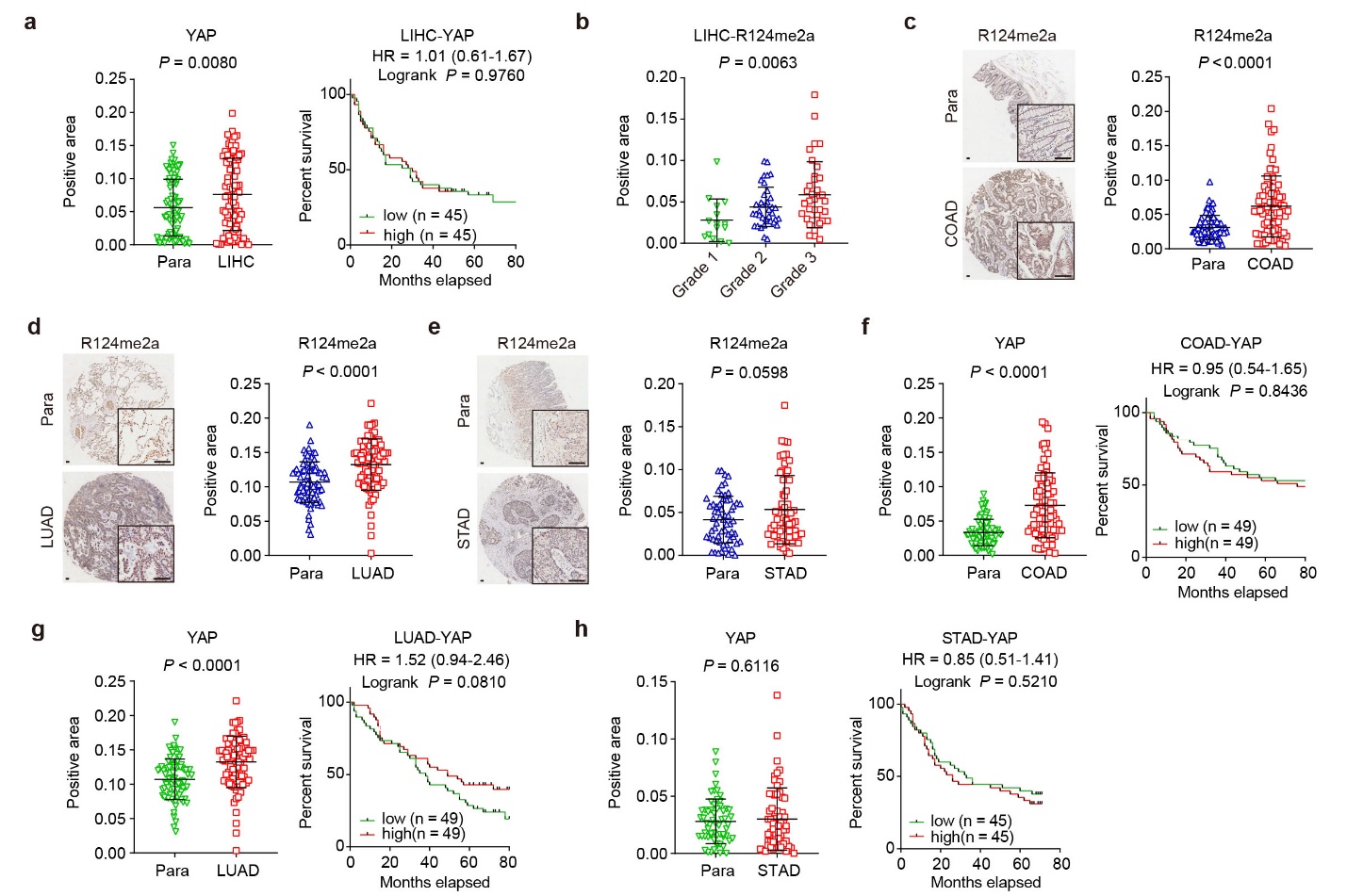


**Supplementary Fig. 7** **YAP-R124me2a is associated with poor patient prognosis for multiple adenocarcinomas. a** Quantification of YAP-positive area in LIHC and para noncancerous tissue (left). Overall survival of LIHC patients according to YAP expression in tumors (right, n = 90). **b** The level of R124me2a in cancer tissues was positively correlated with the grade of malignancy of LIHC (n = 90). **c-e** Representative images of immunohistochemical staining of TMA for R124me2a in COAD (**c**), LUAD (**d**), and STAD (**e**) patients. Scale bar = 100 μm. The quantification of R124me2a-positive area in different tumors and the para noncancerous tissue is shown to the right of each image. **f-h** The quantification of YAP-positive area in COAD (**f**), LUAD (**g**), and STAD (**h**) and para noncancerous tissue (left). Overall survival of patients in TMA according to YAP expression in tumors (right).

**
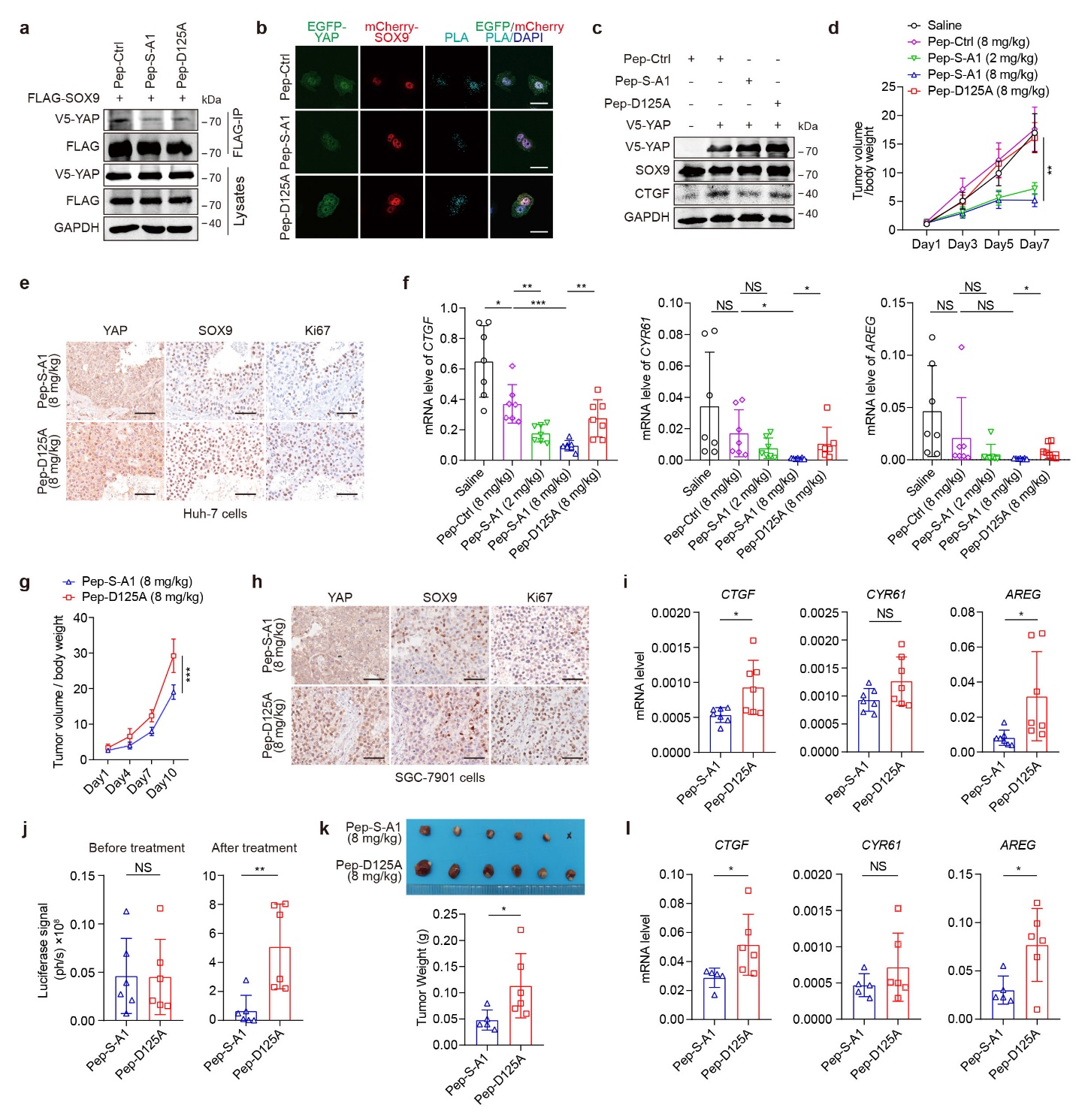
Supplementary Fig. 8 A cell-permeable peptide attenuates YAP function by disrupting the YAP-SOX9 interaction. a** The interaction between V5-YAP and FLAG-SOX9 in Huh-7 cells treated with Pep-Ctrl, Pep-S-A1, or Pep-D125A (10 μM). **b** Representative images of PLA in DOX-induced Huh-7 cells treated with different peptides (10 μM) for 24 h. Scale bar = 20 μm. **c** Immunoblot analysis of SOX9, CTGF, and YAP in Huh-7 cells treated with Pep-Ctrl, Pep-S-A1, or Pep-D125A (10 μM). **d**. Tumor growth was measured in the Huh-7 xenograft model every other day. Data are represented as mean ± SD, ***P* < 0.01. **e** IHC staining of YAP, SOX9, and Ki67 in Huh-7 xenograft tumors treated with Pep-S-A1 (8 mg/kg) or Pep-D125A (8 mg/kg). Scale bar = 100 μm. **f** qPCR analysis of *CTGF, CYR61* and *AREG* expression in Huh-7 xenograft tumors. Data are represented as mean ± SD. **P* < 0.05, ***P* < 0.01, ****P* < 0.001. **g** Tumor growth in the SGC-7901 xenograft model. Data are represented as mean ± SD, ****P* < 0.001. **h** Immunohistochemical staining of YAP, SOX9, and Ki67 in xenograft tumors described in Fig. 6h. Scale bar = 100 μm. **i** qPCR analysis of *CTGF, CYR61* and *AREG* expression in SGC-7901 xenograft tumors. Data are represented as mean ± SD, **P* < 0.05. **j** Statistical analysis of the luciferase activity of mice transplanted with Huh-7 cells before and after peptide delivery. **P* < 0.05. **k** Images (upper) and weight (lower) of tumors isolated from an orthotopic HCC mouse model. **l** qPCR analysis of *CTGF, CYR61* and *AREG* expression in tumors isolated from an orthotopic HCC mouse model. Data are represented as mean ± SD, NS, not significant, **P* < 0.05.
