# Supplementary material for: SRY-Box transcription factor 9 triggers YAP nuclear entry via direct interaction in tumors
Source: Signal Transduct Target Ther. 2024 Apr 24;9:96. doi: 10.1038/s41392-024-01805-4 (PMC11039692; doi:10.1038/s41392-024-01805-4)

Original and uncropped films of Western blots for **main text Figures.**

Fig.1


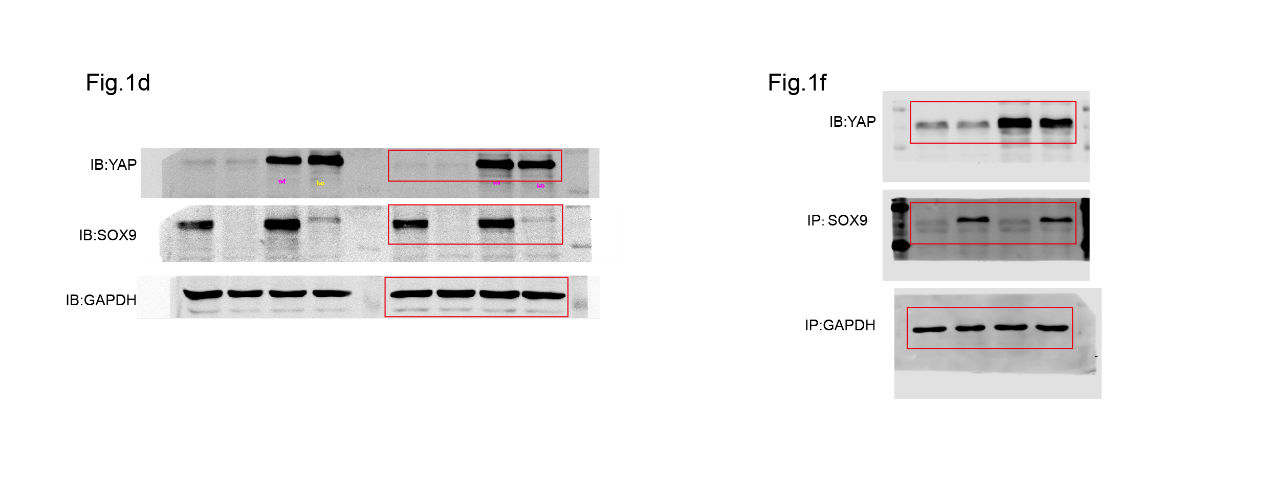


Fig.2
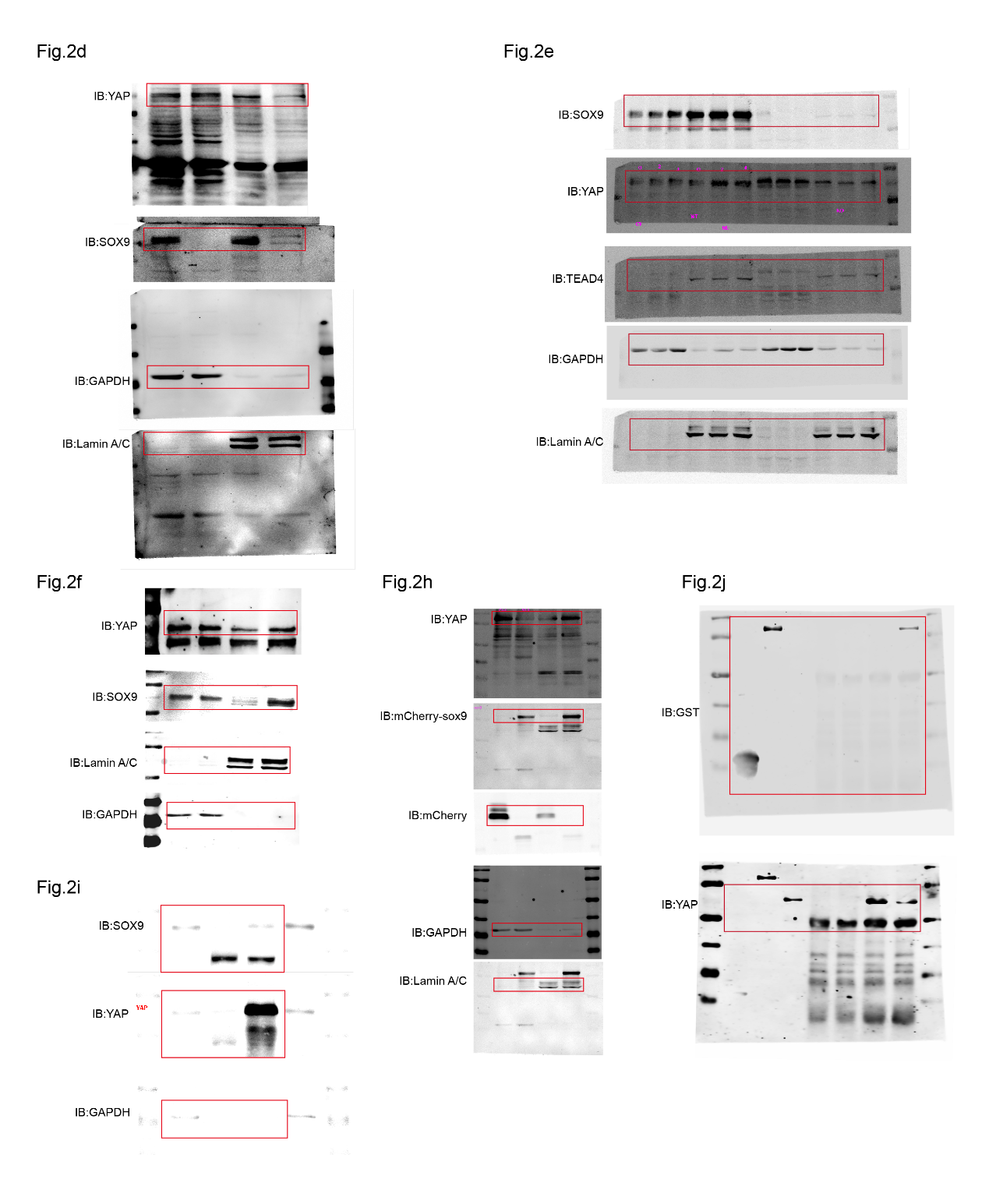


Fig.3
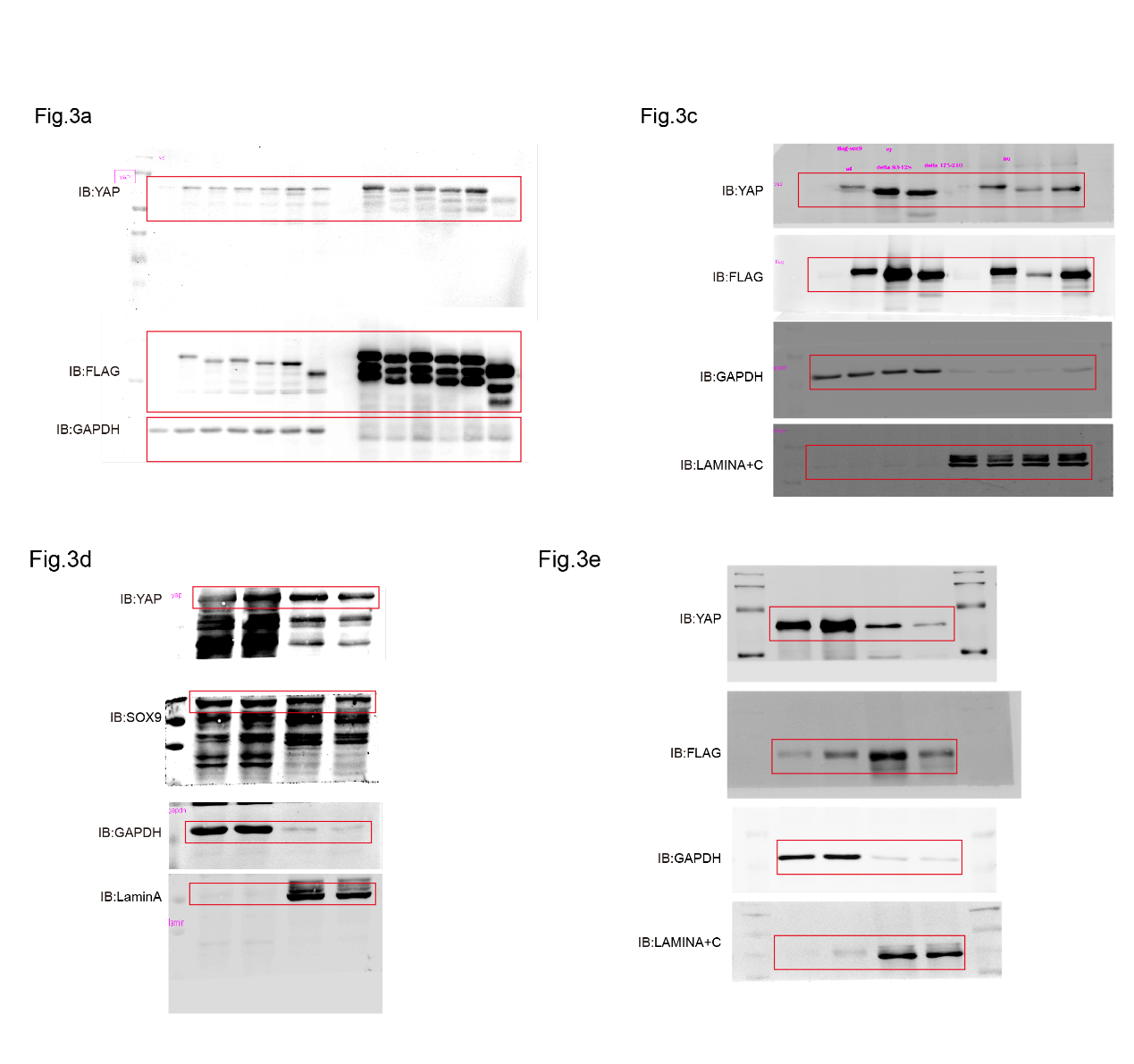


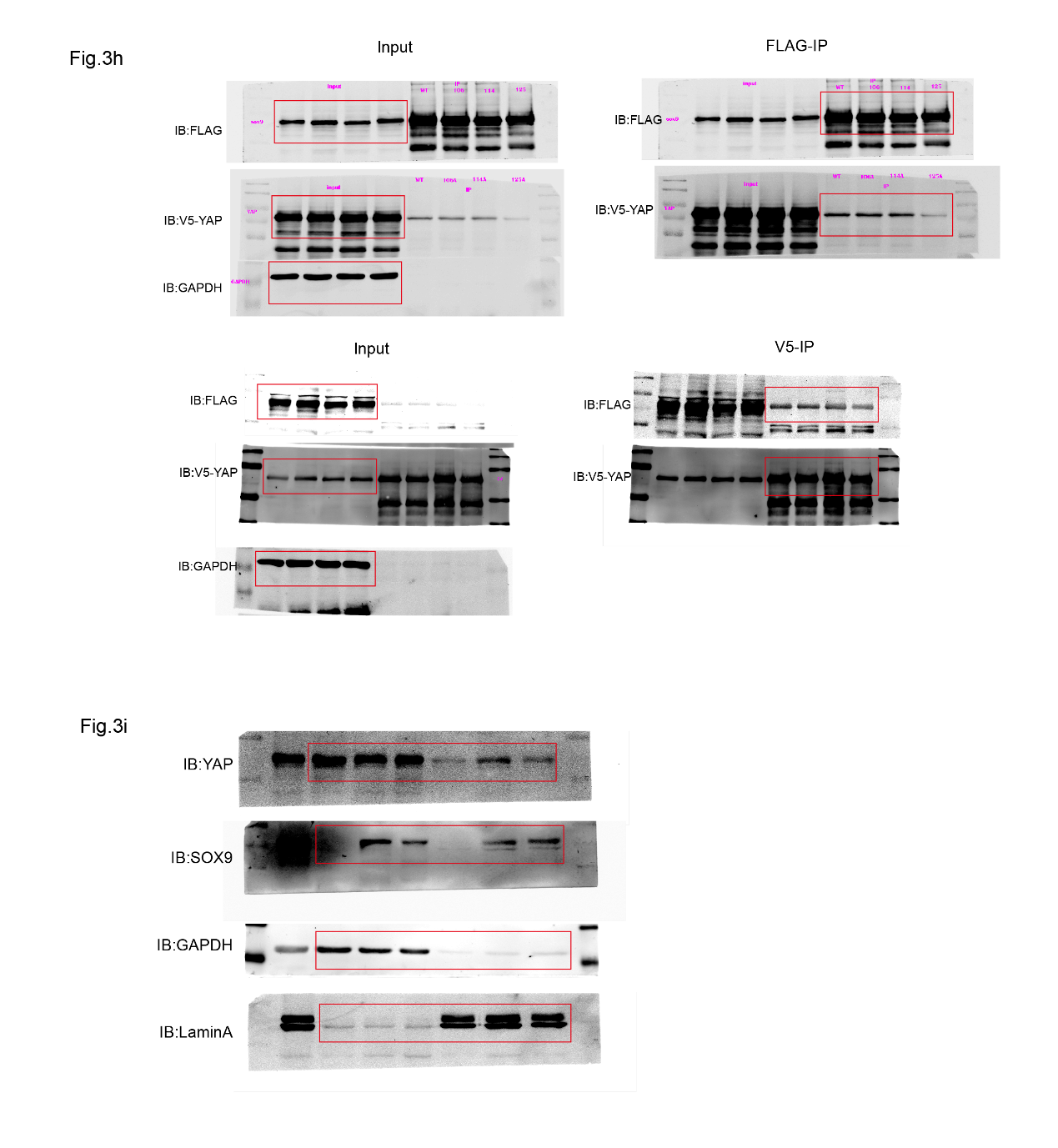


Fig.4


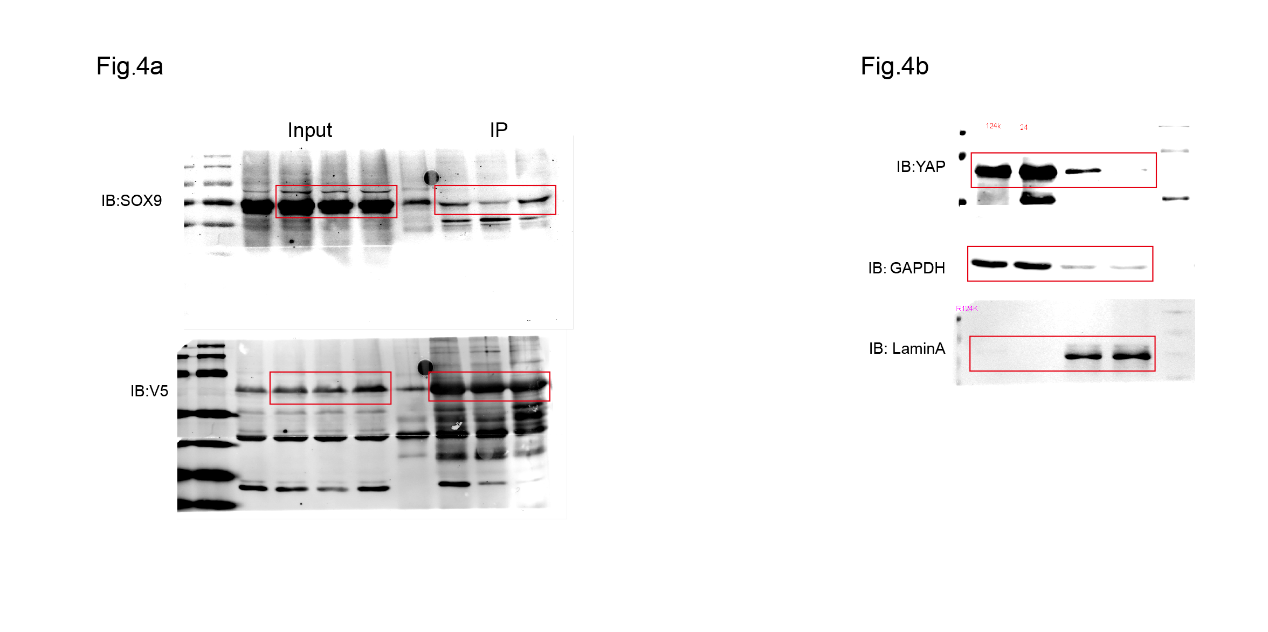


Fig.5­­­­

­­
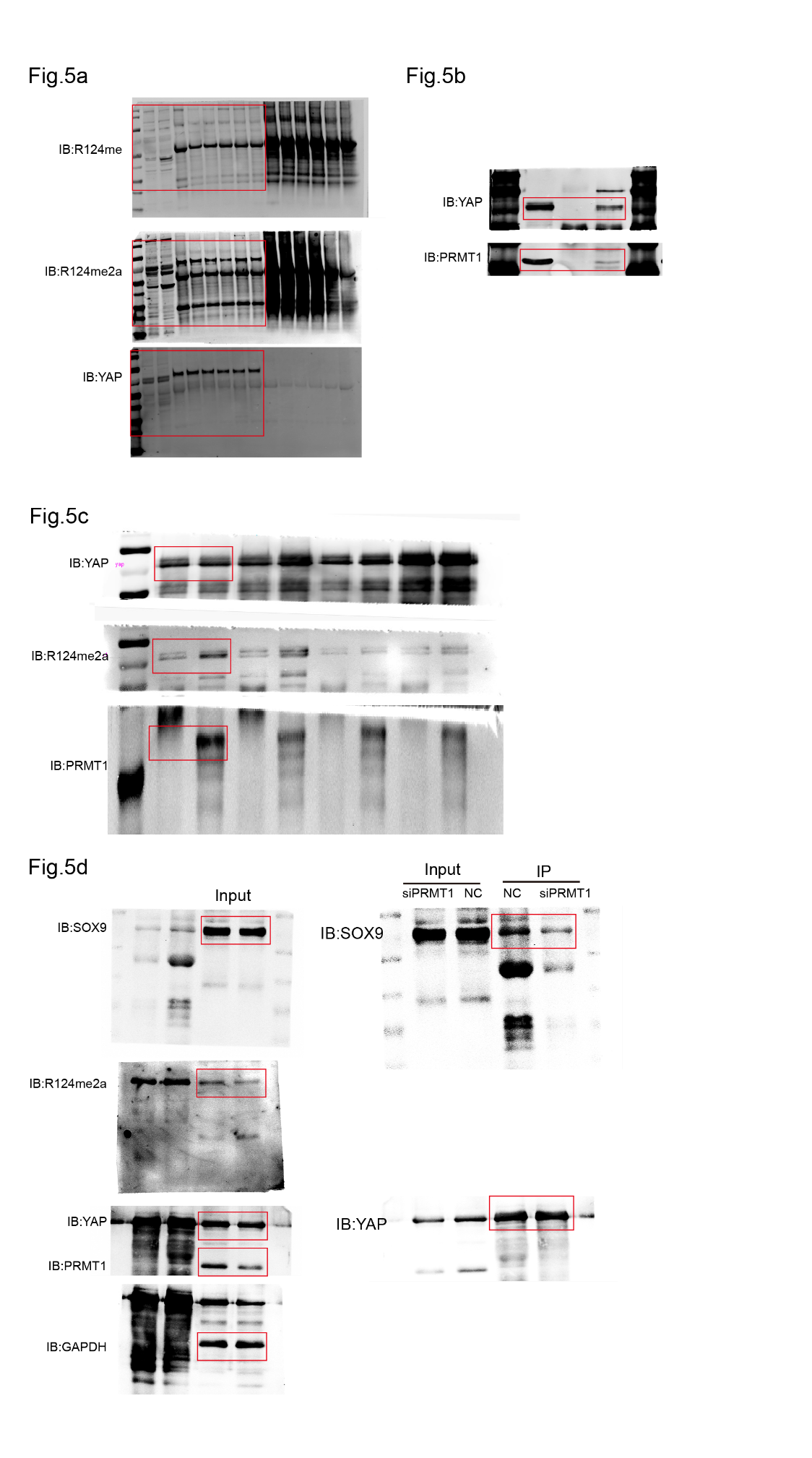


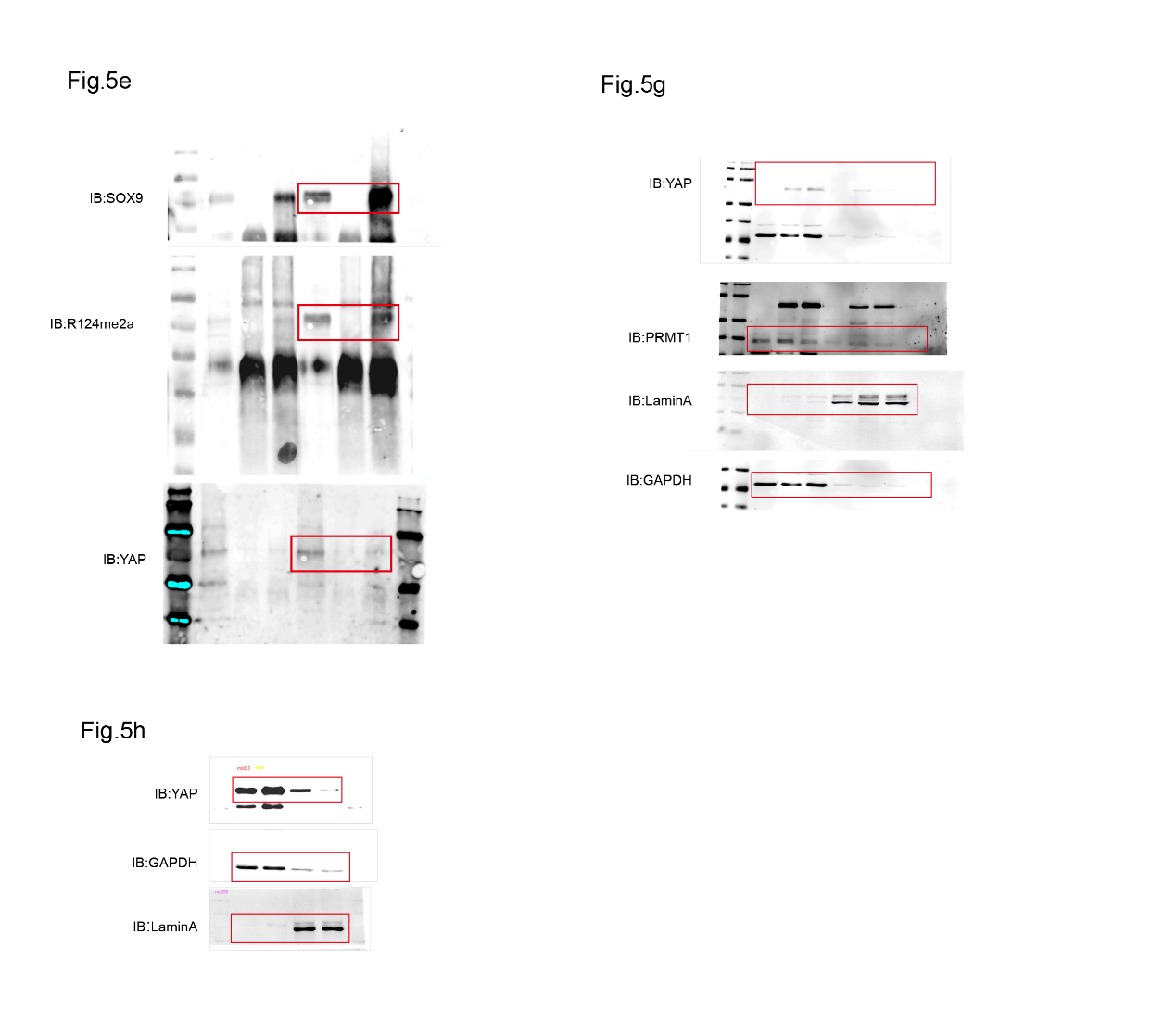


Fig.6


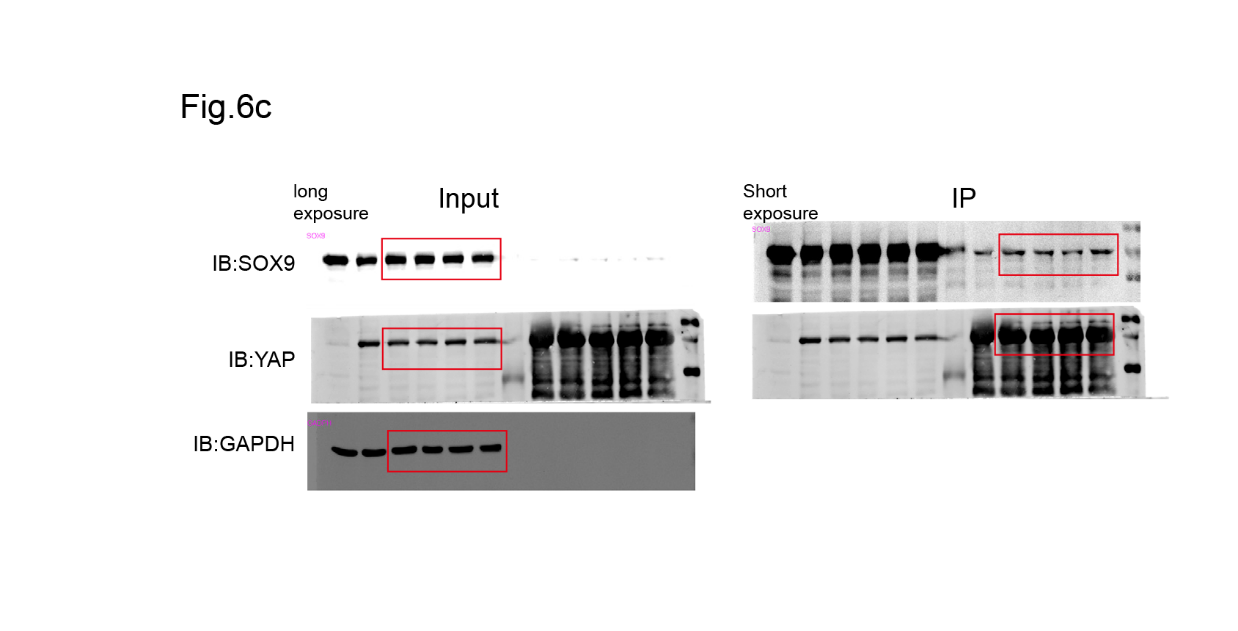


Original and uncropped films of Western blots for **supplementary Figures.**

Supplementary Fig.1


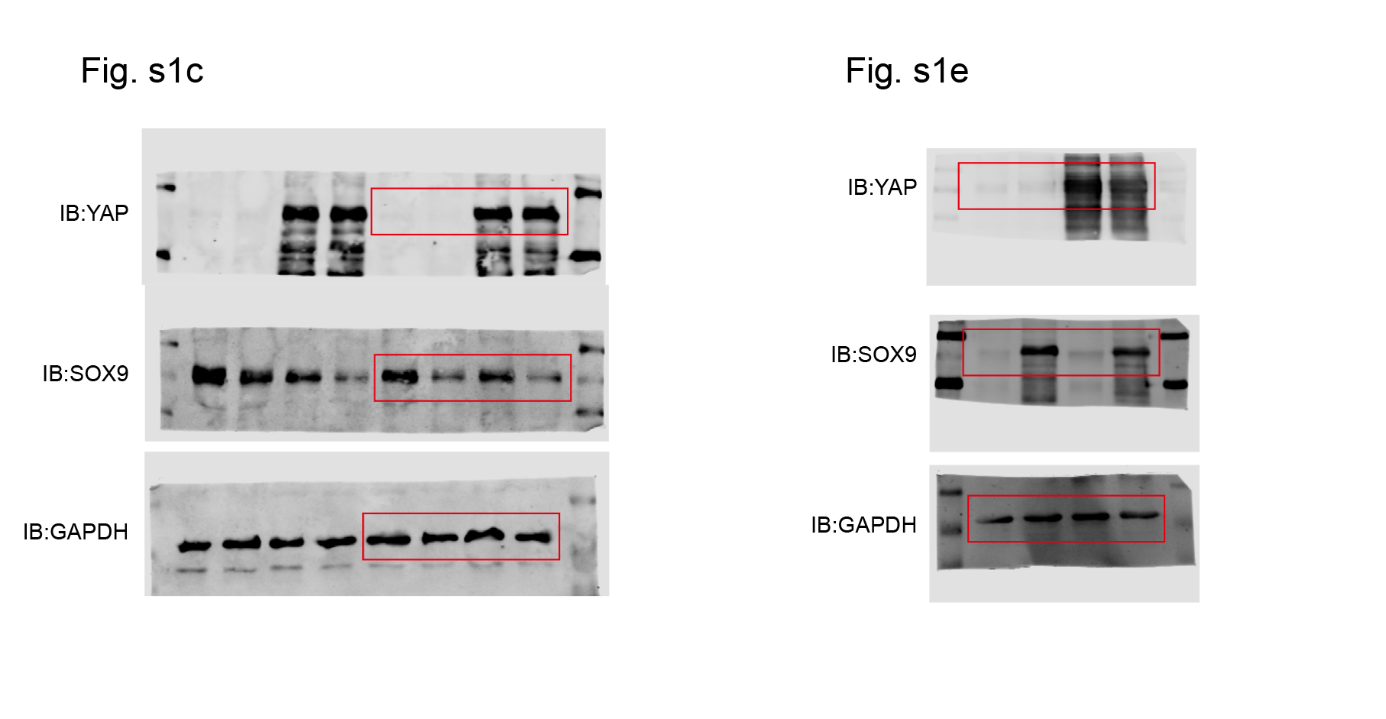


Supplementary Fig.2


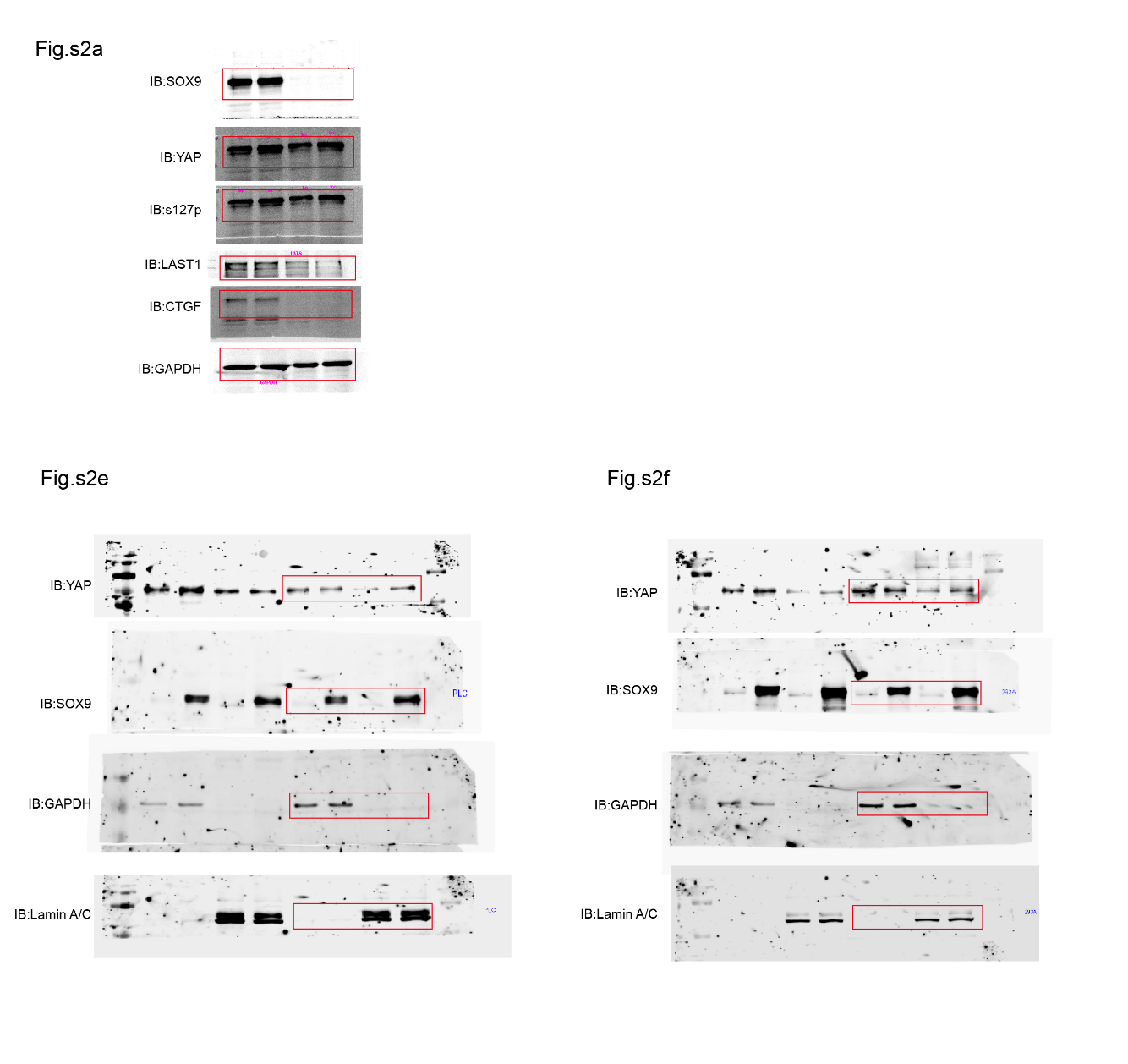


Supplementary Fig.3


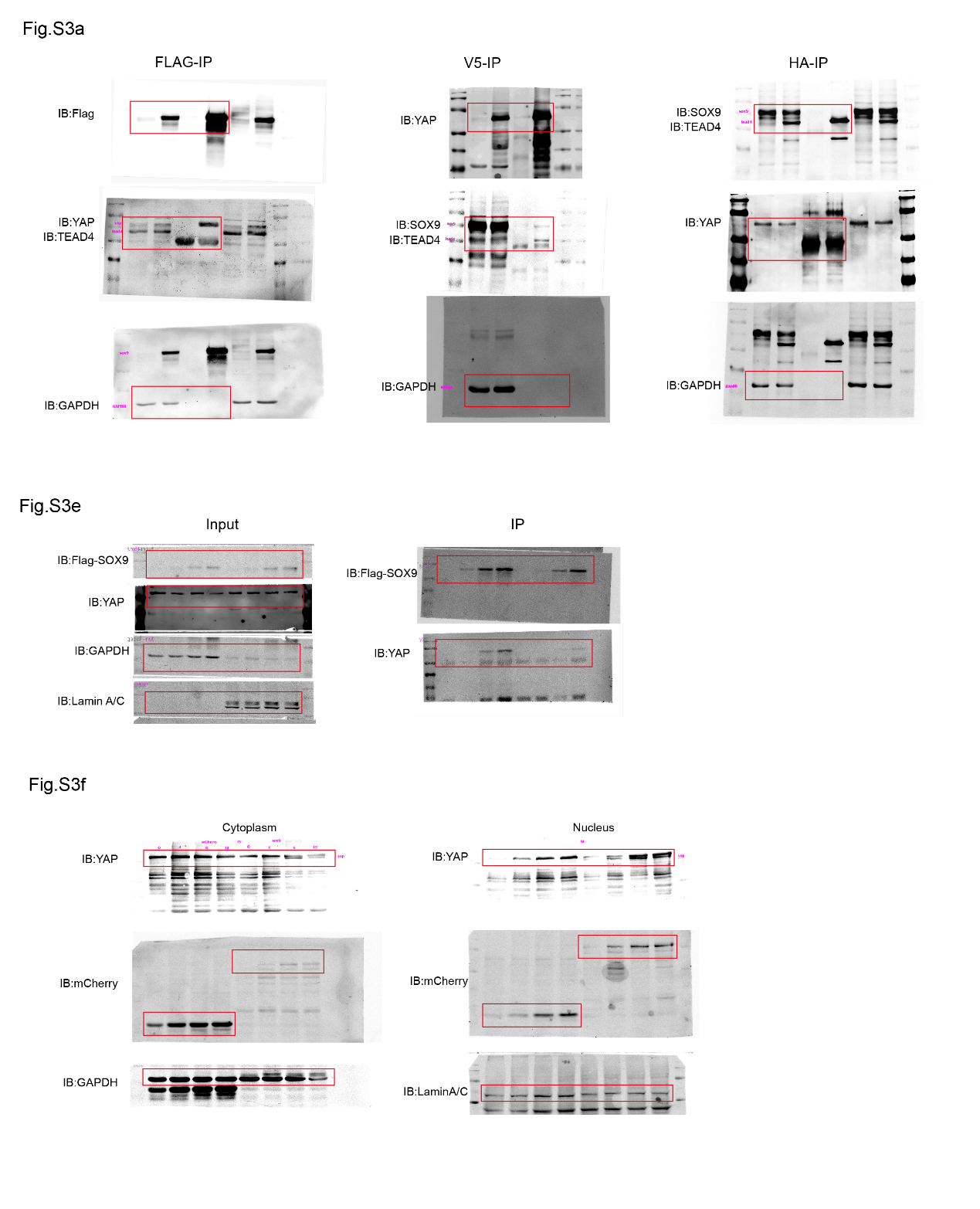


Supplementary Fig.4


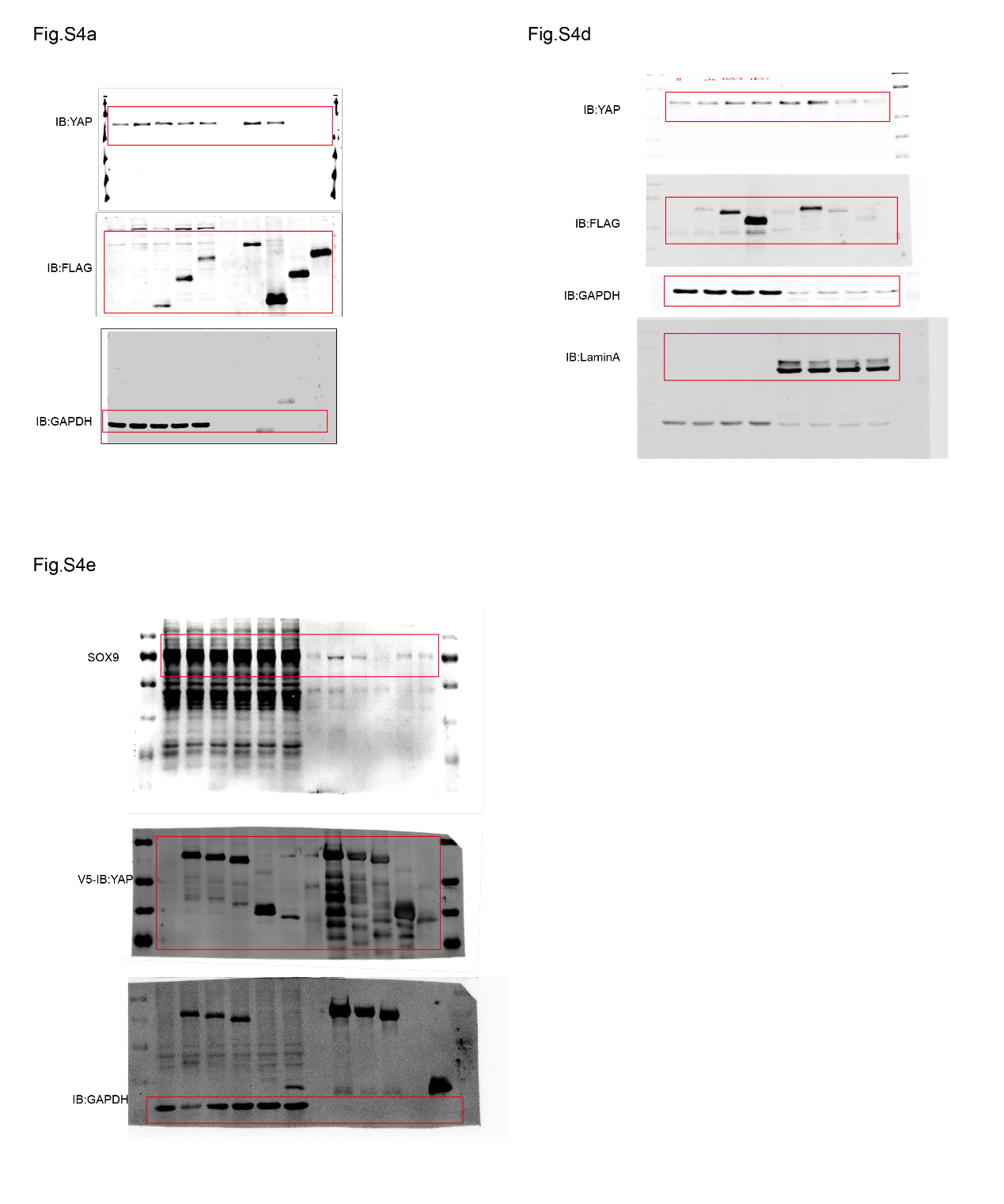


Supplementary Fig.5


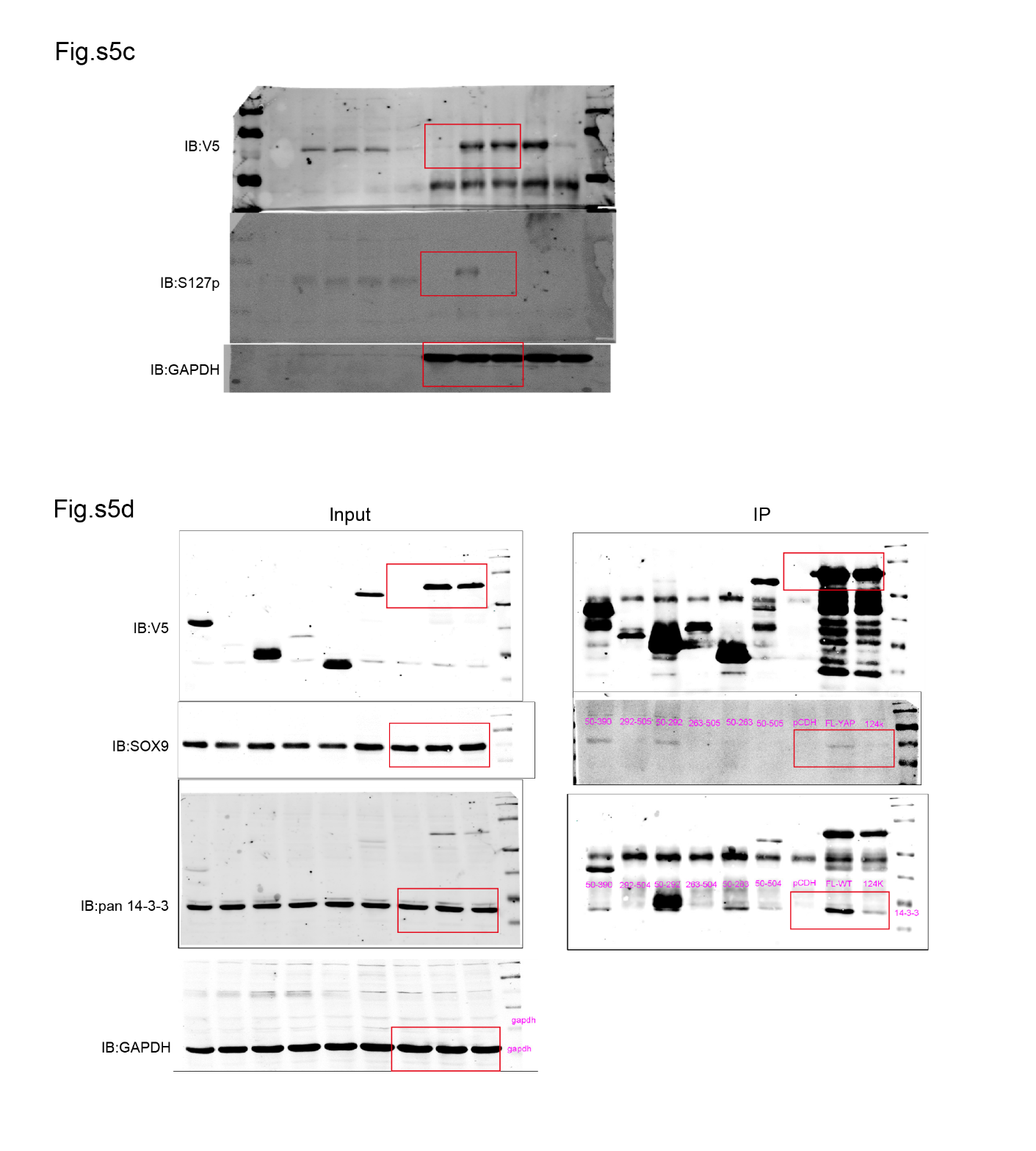


Supplementary Fig.6


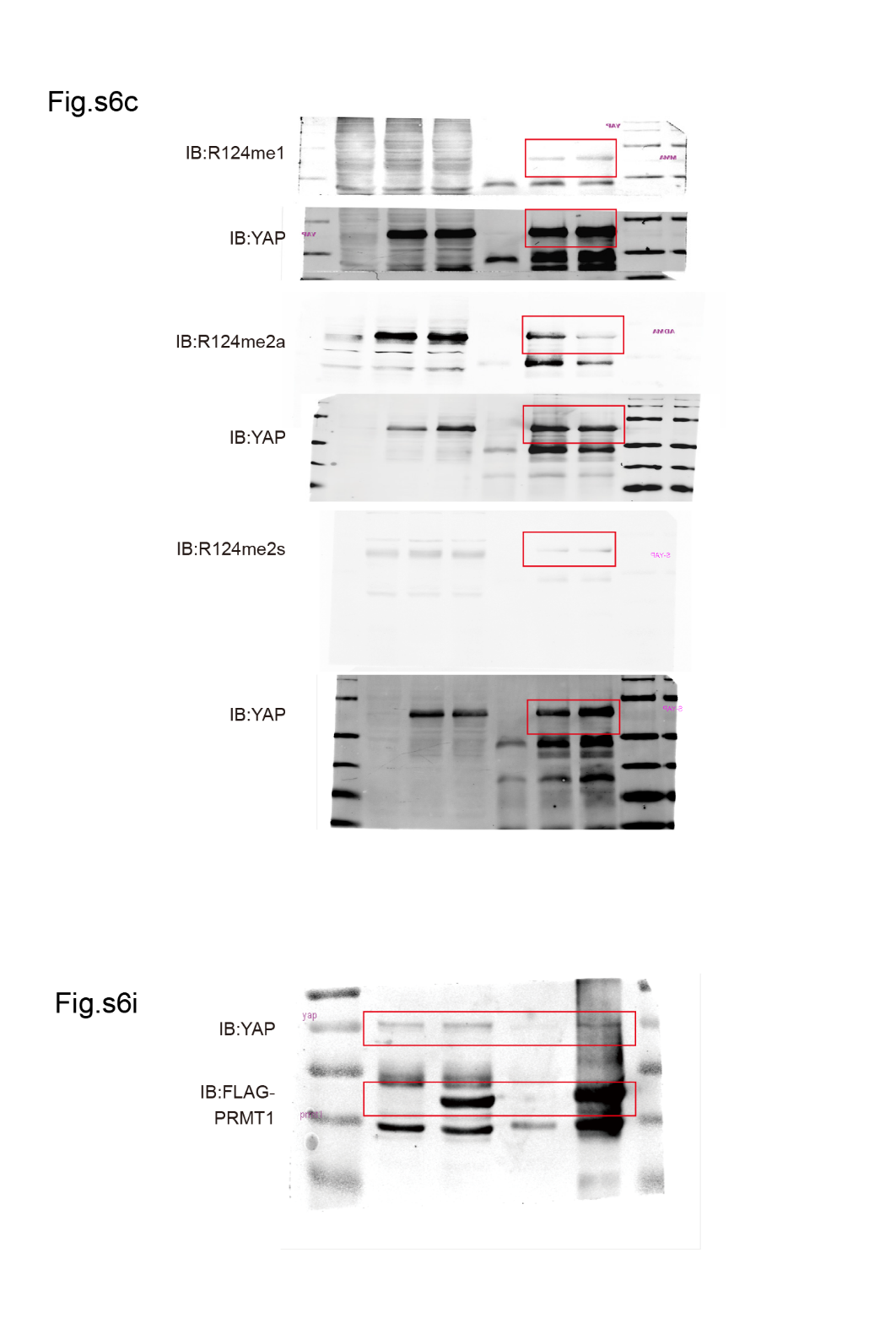


Supplementary Fig.8


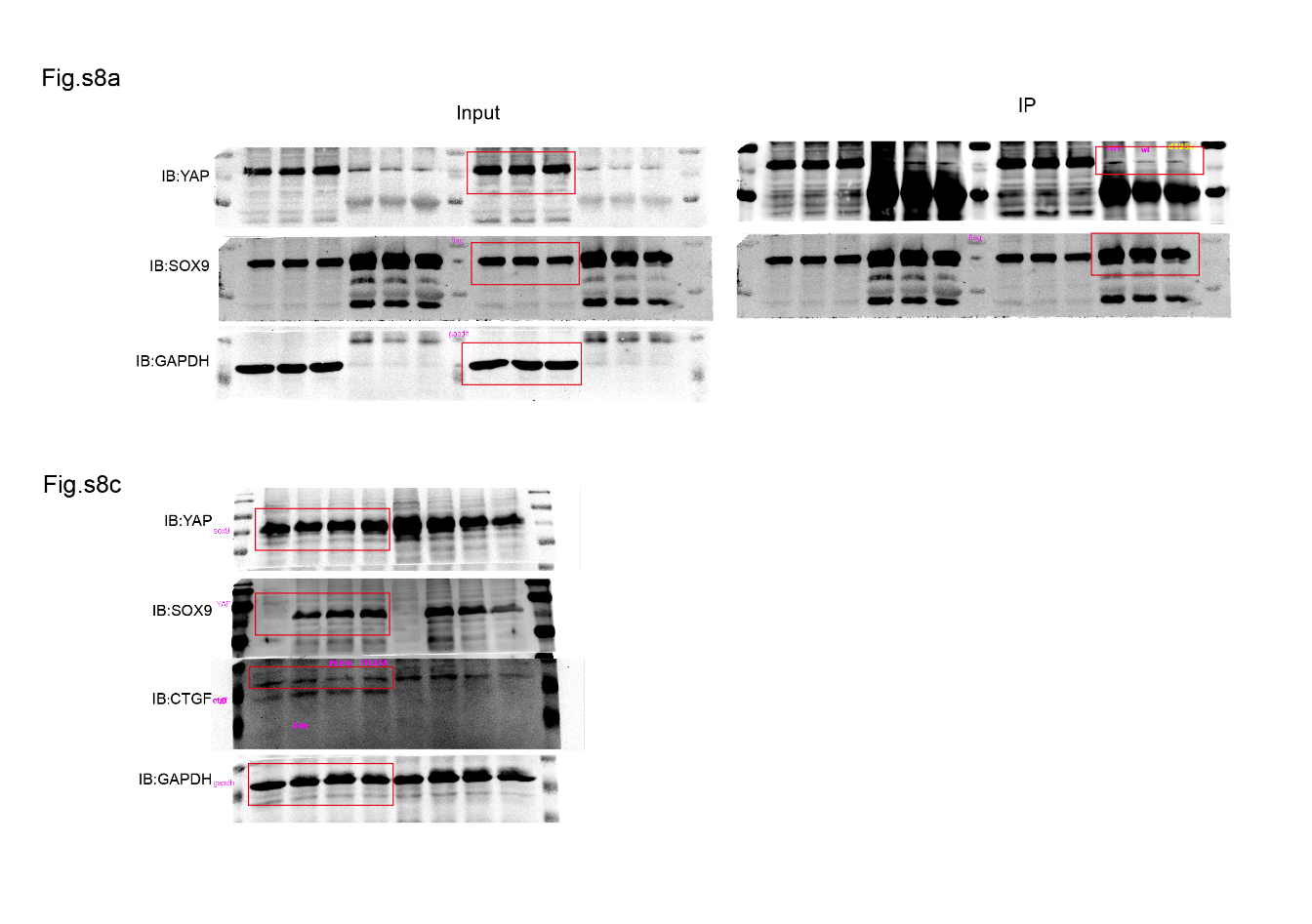

Supplement: Supplementary file 2 — original western blot figures [file 41392_2024_1805_MOESM2_ESM.docx]
