# Supplementary material for: SRY-Box transcription factor 9 triggers YAP nuclear entry via direct interaction in tumors
Source: Signal Transduct Target Ther. 2024 Apr 24;9:96. doi: 10.1038/s41392-024-01805-4 (PMC11039692; doi:10.1038/s41392-024-01805-4)
Supplement: Supplementary file 3 — Supplementary Table 1 [file 41392_2024_1805_MOESM3_ESM.pdf]

Supplementary table 1: The peptide sequence used in this paper

Peptide	Sequence	Source
R124 unmodified peptide	AGALTPQHVRAHSSPASLQL	Chinapeptides
R124 asymmetric methylated peptide	AGALTPQHVR(me2a)AHSSPASLQL	Chinapeptides
R124 mono methylated peptide	AGALTPQHVR(me)AHSSPASLQL	Chinapeptides
R124 symmetric methylated peptide	AGALTPQHVR(me2s)AHSSPASLQL	Chinapeptides
R124K peptide	AGALTPQHVKAHSSPASLQL	Chinapeptides
SOX9 94-126 (WT)	RVNGSSKNKPHVKRPMNAFMVWAQAARRKLADQ	Chinapeptides
SOX9 94-126 (D125A)	RVNGSSKNKPHVKRPMNAFMVWAQAARRKLAAQ	Chinapeptides
