# Supplementary material for: SRY-Box transcription factor 9 triggers YAP nuclear entry via direct interaction in tumors
Source: Signal Transduct Target Ther. 2024 Apr 24;9:96. doi: 10.1038/s41392-024-01805-4 (PMC11039692; doi:10.1038/s41392-024-01805-4)
Supplement: Supplementary file 4 — Supplementary Table 2 [file 41392_2024_1805_MOESM4_ESM.pdf]

Supplementary table 2: The oligonucleotides used in this paper

Oligonucleotides	forward	reverse
siNC	UUCUCCGAACGUGUCACGUtt	
siPRMT1#1	GAGUUCACACGCUGCCACAtt	
siSOX9	CCACCUUCACCUACAUGAAtt	
Mouse Gapdh RT-PCR	F: ACACATTGGGGGTAGGAACA	R: AACTTTGGCATTGTGGAAGG
Mouse Yap RT-PCR	F: AGGAGAGACTGCGGTTGAAA	R: CCTGAGACATCCCAGGAGAA
Mouse Ctgf RT-PCR	F: GGCCAAATGTGTCTTCCAGT	R: CAAAGCAGCTGCAAATACCA
Mouse Cyr61 RT-PCR	F: GGAACCGCATCTTCACAGTT	R: CAAAGCAGCTGCAAATACCA
Mouse Areg RT-PCR	F: GGCAGTGCATGGATTCTTTT	R: CTGGCAGTGAACTCTCCACA
Human GAPDH RT-PCR	F: TTGATTTTGGAGGGATCTCG	R: GAGTCAACGGATTTGGTCGT
Human YAP RT-PCR	F: CAGCAACTGCAGATGGAGAA	R: TGGATTTTGAGTCCCACCAT
Human CTGF RT-PCR	F: GTAATGGCAGGCACAGGTCT	R: CCGTACTCCCAAAATCTCCA
Human CYR61 RT-PCR	F: GGTCAAAGTTACCGGGCAGT	R: GGAGGCATCGAATCCCAGC
Human OPN RT-PCR	F: TGAAACGAGTCAGCTGGATG	R: TGAAATTCATGGCTGTGGAA
Human COL2A1 RT-PCR	F: GGGAGTAATGCAAGGACCAA	R: ATCATCACCAGGCTTTCCAG
Human AREG RT-PCR	F: GCTGTCGCTCTTGATACTCG	R: ACGCTTCCCAGAGTAGGTGT
