# Supplementary material for: SRY-Box transcription factor 9 triggers YAP nuclear entry via direct interaction in tumors
Source: Signal Transduct Target Ther. 2024 Apr 24;9:96. doi: 10.1038/s41392-024-01805-4 (PMC11039692; doi:10.1038/s41392-024-01805-4)
Supplement: Supplementary file 5 — Supplementary Table 3 [file 41392_2024_1805_MOESM5_ESM.pdf]

Supplementary table 3: The antibodies used in this paper

Antibodies	Source	Indentifier
Mouse monoclonal anti-V5	Abcam	Cat# ab27671
Rabbit monoclonal anti-FLAG	Sigma	Cat#F2555
Rabbit monoclonal anti-PRMT1	Abcam	Cat# ab190892
Mouse monoclonal anti-PRMT1	Santa Cruz	Cat# sc-166963
Rabbit monoclonal anti-active YAP	Abcam	Cat# ab205270
Mouse monoclonal anti-YAP	CST	Cat# 12395
Rabbit monoclonal anti-YAP	CST	Cat# 14074
Rabbit monoclonal anti-Phospho-YAP (Ser127)	CST	Cat# 13008
Rabbit polyclonal anti-Me2a-YAP (Arg124)	Chinapeptides	N/A
Rabbit monoclonal anti-LATS1	CST	Cat# 3477
Rabbit monoclonal anti-SOX9	Abcam	Cat# ab185230
Rabbit polyclonal anti-SOX9	Millipore	Cat# AB5535
Mouse monoclonal anti-GFP	Santa Cruz	Cat#SC9996
Rabbit polyclonal anti-mCherry	Abcam	Cat#ab183628
Rabbit polyclonal anti-CTGF	Abcam	Cat# ab6992
Rabbit monoclonal anti-HA-tag	CST	Cat# 3724
Rabbit monoclonal anti-GST-tag	Beyotime	Cat#AF2299
Mouse monoclonal anti-pan-14-3-3	Santa Cruz	Cat# sc-1657
Rabbit polyclonal anti-TEAD4	Abcam	Cat# ab151274
Rabbit monoclonal anti-AFP	MaiXin	RMA-1069
Rabbit monoclonal anti-GPC3	MaiXin	RMA-0667
Rabbit polyclonal anti-CK19	Abbomax	602-670
Mouse monoclonal anti-GAPDH	CST	Cat# 97166
Rabbit polyclonal anti-Ki67	Abcam	Cat# ab15580
